# Machine learning approach for predicting cardiovascular disease in Bangladesh: evidence from a cross-sectional study in 2023

**DOI:** 10.1186/s12872-024-03883-2

**Published:** 2024-04-18

**Authors:** Sorif Hossain, Mohammad Kamrul Hasan, Mohammad Omar Faruk, Nelufa Aktar, Riyadh Hossain, Kabir Hossain

**Affiliations:** 1 Department of Statistics, Noakhali Science and Technology University, Noakhali, 3814 Bangladesh; 2 Department of Information and Communication Engineering, Noakhali Science and Technology University, Noakhali, 3814 Bangladesh

**Keywords:** Cardiovascular disease, Machine learning, Random forest, Feature selection, Bangladesh

## Abstract

**Background:**

Cardiovascular disorders (CVDs) are the leading cause of death worldwide. Lower- and middle-income countries (LMICs), such as Bangladesh, are also affected by several types of CVDs, such as heart failure and stroke. The leading cause of death in Bangladesh has recently switched from severe infections and parasitic illnesses to CVDs.

**Materials and methods:**

The study dataset comprised a random sample of 391 CVD patients' medical records collected between August 2022 and April 2023 using simple random sampling. Moreover, 260 data points were collected from individuals with no CVD problems for comparison purposes. Crosstabs and chi-square tests were used to determine the association between CVD and the explanatory variables. Logistic regression, Naïve Bayes classifier, Decision Tree, AdaBoost classifier, Random Forest, Bagging Tree, and Ensemble learning classifiers were used to predict CVD. The performance evaluations encompassed accuracy, sensitivity, specificity, and area under the receiver operator characteristic (AU-ROC) curve.

**Results:**

Random Forest had the highest precision among the five techniques considered. The precision rates for the mentioned classifiers are as follows: Logistic Regression (93.67%), Naïve Bayes (94.87%), Decision Tree (96.1%), AdaBoost (94.94%), Random Forest (96.15%), and Bagging Tree (94.87%). The Random Forest classifier maintains the highest balance between correct and incorrect predictions. With 98.04% accuracy, the Random Forest classifier achieved the best precision (96.15%), robust recall (100%), and high F1 score (97.7%). In contrast, the Logistic Regression model achieved the lowest accuracy of 95.42%. Remarkably, the Random Forest classifier achieved the highest AUC value (0.989).

**Conclusion:**

This research mainly focused on identifying factors that are critical in impacting patients with CVD and predicting CVD risk. It is strongly advised that the Random Forest technique be implemented in a system for predicting cardiac diseases. This research may change clinical practice by providing doctors with a new instrument to determine a patient’s CVD prognosis.

**Supplementary Information:**

The online version contains supplementary material available at 10.1186/s12872-024-03883-2.

## Introduction

Cardiovascular diseases (CVD) encompass several issues affecting the cardiopulmonary system and veins. These include various types of malignancies, cardiac failure (HF), cerebrovascular disorders such as stroke, and coronary illnesses such as heart attack [1]. CVDs constitute a broad category of cardiac and blood vessel conditions, including coronary artery disease, which is characterized by insufficient oxygenated blood supply to the heart and cardiovascular illness, impacting blood circulation in the cerebellum. Additionally, chronic heart failure is a condition in which the heart lobes suffer permanent damage [2].

CVDs encompasses a range of disorders that affect the heart and blood vessels. This category includes conditions, such as coronary heart disease, cerebrovascular disease, rheumatic heart disease, and other related ailments. According to the World Health Organization (WHO), approximately 17.9 million deaths occurred due to CVD worldwide in 2016, accounting for 31% of all deaths worldwide. Among these deaths, 85% were due to heart failure [3]. Heart disease occurs when the heart fails to circulate enough blood to organs. It is frequently caused by high blood pressure, insulin resistance, infections, or other cardiovascular disorders [4].

CVD is a major health issue worldwide, affecting approximately 26 million individuals globally each year [5]. Individuals in lower- and middle-income countries (LMICs) such as Bangladesh are affected by several types of CVDs [6]. The leading cause of death in Bangladesh has increasingly switched from severe infections and parasitic illnesses to CVDs, accounting for only 8% of total deaths in 1986, which was reduced to 5% in 2018, with a higher prevalence in urban areas (8%) than in rural areas (2%) [6, 7]. In Bangladesh, heart disease had the highest reported prevalence (21%), whereas stroke had the lowest recorded prevalence (1%) in 2018 [7].

According to previous studies, the most important behavioral risk factors for CVDs, particularly heart disease and stroke, are unhealthy diet, physical inactivity, tobacco use, and harmful use of alcohol [8]. Dyslipidemia, tobacco use, diabetes, hypertension, and overweight have also been reported as potential risk factors for heart failure in previous studies [9, 10]. Another study conducted by Hossain et al. (2023) found that age, sex, smoking, obesity, diet, physical activity, stress, chest pain type, previous chest pain, diastolic blood pressure, diabetes, and troponin were the most important factors for identifying CVD risk [11]. Different experiences at different stages of epidemiological transition and urbanization, with varying life expectancies, diverse demographic profiles, and differences in environmental and genetic risk factors, could explain the different relationships between these risk factors and CVD mortality in Asian and Western societies [12].

Patients with heart disease do not exhibit symptoms in the early stages of the disease, but they do in later stages, which can often be too late to manage or treat [13]. As a result, despite the difficulty, early detection and prediction of CVD hypersensitivity in seemingly healthy patients is essential for determining the prognosis [13]. It remains difficult for cardiologists to diagnose and treat patients in their early stages [14]. Working with patient databases for patients with heart disease is a practical application. Therefore, it is reasonable to consider using the knowledge of diverse professionals compiled in databases to aid in the diagnosis process [15]. Every conventional model for assessing CVD risk implicitly assumes that every risk factor is linearly related to the CVD outcome [14]. Several risk factors with nonlinear interactions are among the complicated linkages that these models tend to oversimplify [14]. Prediction models based on machine learning algorithms are robust against common limitations such as nonlinearity, multicollinearity, interaction, and complexities available in large datasets in traditional statistical models [16]. Moreover, it is envisaged that prediction models based on machine-learning algorithms demonstrate better predictive performance than traditional statistical methods [16]. For this reason, machine learning approaches have shown great promise in supporting clinical decision-making, helping create clinical guidelines and management algorithms, and encouraging the adoption of clinical practices based on evidence for the treatment of cardiovascular diseases (CVDs) [13]. Additionally, the early diagnosis of CVDs using machine learning approaches can lessen the need for costly and time-consuming clinical and laboratory tests, which will save costs for both individuals and the healthcare system [13].

Recently, machine learning models have been widely used to precisely predict CVD risk factors. Hossain et al. (2023) analyzed a study 2023 to predicting the risk of heart failure using distinct artificial intelligence techniques (logistic regression, Naïve Bayes, K-nearest neighbor (K-NN), support vector machine (SVM), decision tree, random forest, and multilayer perceptron (MLP) [11]. In this study, the authors found that the Random Forest model achieved the highest accuracy rate (90%) compared to other machine learning models. Furthermore, previous studies have used a machine learning approach to predict heart failure risk using clinical, behavioral, socio-demographic, and socioeconomic features [17, 18]. Ensemble learning is critical for producing excellent forecast outcomes in a variety of real-world applications. For example, ensemble machine learning technologies such as random forests, XGBoost, light gradient boosting machines, and Soft Voting have improved the early identification of diabetes mellitus by merging numerous models to increase predictive accuracy. Their efficiency and cost-effectiveness make them excellent instruments for diabetes screening and diagnosis, providing faster and less expensive alternatives to traditional procedures [19]. In the field of health research, ensemble learning methods, such as bagging, boosting, and stacking, are used to increase the accuracy and reliability of Alzheimer's disease detection models by mixing several machine learning algorithms [20]. According to research in the field of sports science, footballer positions may be reliably and precisely classified with high accuracy when stacked ensemble machine learning models are applied to datasets, such as FIFA'19 [21]. A novel hybrid data-mining approach predicts Salmonella prevalence in agricultural waterways by combining ensemble feature selection and machine learning methods. The combined ANN and RF ensemble outperformed existing approaches, providing an enhanced strategy for accurately detecting and mitigating agricultural water sources [22]. In forecasting Escherichia coli levels in agricultural water, ensemble models such as random forest and AdaBoost using meteorological data performed better than individual models, indicating the potential for more precise predictions in agricultural contexts [23]. In addition, Long Short-Term Memory (LSTM) has been effectively used for cryptocurrency data analysis, with remarkable success in accurately anticipating price patterns and providing useful insights for investors and traders in the unpredictable crypto market [24].

A recent literature review showed that some model performances, but lack reproducibility, suffer some problems and limit their reliability [25], [26], [27], [28]. Some models have been established recently to improve model effectiveness, but they still do not show optimal performance [29], [30], [31], [32]. To address this gap, this study was conducted to learn more about the prevalence and risk factors of cardiac disease in Bangladesh. Therefore, this study seeks to respond to the following research questions, considering the study's aims and objectives:

- To accurately predict cardiovascular diseases (CVD) using different machine learning and ensemble learning approaches
- To identify significant predictors of heart failure.
- To determine better classification technique among applicable model’s cardiovascular diseases (CVD) predicting

This study compares multiple modeling strategies, including logistic regression, Naïve Bayes classifier, Decision Tree, AdaBoost classifier, Random Forest, Bagging Tree, and Ensemble learning classifiers, to reliably predict cardiovascular diseases (CVD). First, we describe these methods to demonstrate their usefulness and optimization methodologies. Next, we divided the completed preprocessed datasets into training and test sets for model building and forecasting, along with performance assessment parameters, including accuracy, precision, recall, and F1 score. Finally, the chosen models were used to properly diagnose heart failure, followed by an evaluation of their CVD prediction ability. This study could assist physicians and health scientists in classifying high-risk patients and in making a novel diagnosis to prevent cardiac failure using counseling and medicines.

## Methods

### Data collection

Bangladeshi individuals aged > 15 years were included in this study. In this study, individuals with and without cardiac disease were considered. A questionnaire was used to collect primary data from Dhaka Medical College, the National Institute of Cardiovascular Disease (NICVD), and BIRDEM. These three institutions provide treatment for patients with cardiovascular disease. Patients from all regions of Bangladesh were included in this study. The research dataset comprised a random sample of clinical reports of 391 patients with cardiac failure gathered from August 2022 to April 2023. In addition, 260 data points were also collected from individuals with no cardiac failure problems for comparison purposes. The sample size was estimated using Cochran's law, and data were gathered using a simple random sampling procedure [33].

### Dependent variables

In this study, we considered cardiac disease as a dependent variable, with and without cardiac disease. We asked patients, Do you have a heart disease according to the diagnosis? and reported answers of ‘yes’ or’ no.

### Independent variables

In our study, we considered several types of independent variables including gender (Male, Female), education (No education, primary, secondary, higher secondary), division (Dhaka, Chattogram, Khulna, Rajshahi, Barisal, Sylhet, Mymensingh, Rangpur), residence (urban, rural), socio-economic status (< 20,000, 20,000–40000, > 40,000 Taka), take physical exercises regularly (yes, no), Consume two or more serving of fruits or vegetables per day (yes, no), eat junk food regularly (yes, no), Keep too much salt in your diet (yes, no), feel bad about yourself (yes, no), Feel no interest or pleasure in doing any things (yes, no), Feel hopeless (yes, no), have sound sleep at night (yes, no), Have smoking habit (yes, no), Have the habit of drinking alcohol (yes, no), Have blood pressure (yes, no), Have the presence of high cholesterol level (yes, no), Have any family history of heart failure disease (yes, no), Have the presence of anemia (yes, no), Have any type of diabetes (yes, no), Have the presence of hypertension (yes, no), Have sleep apnea problem (yes,no), Have irregular heart rhythms (yes, no), Have coronary artery disease (yes, no), Have angina symptoms (yes, no), Have kidney, lungs or other major disease (yes, no), Take statin to decrease cholesterol level (yes, no), BMI (calculated from height and weight), and platelets, creatinine and sodium levels are considered as independent variables for this study. For further clarification, please see the questionnaire attached in a supplementary file (see Table 1).

**Table 1:** Descriptive statistics (categorical) of different variables of Cardiovascular patient

Variables	Category	n (%)
Cardiovascular disease	No Yes	260(39.9) 391(60.1)
Gender	Female Male	241(37.0) 410(63.0)
Education	No education Primary Secondary Higher secondary	111(17.1) 250(38.4) 173(26.6) 117(18.0)
Division	Dhaka Chattogram Khulna Rajshahi Barisal Sylhet Mymenshing Rangpur	128(19.7) 142(21.8) 59(9.1) 54(8.3) 69(10.6) 60(9.2) 88(13.5)
Socio-economic status	< 20,000 20,000–40,000 > 40,000	139(21.4) 383(58.8) 129(19.8)
Residence	Urban Rural	254(39.0) 397(61.0)
Take physical exercise regularly	No Yes	213(32.7) 438(67.3)
Have sound sleep at night	No Yes	287(44.1) 364(55.9)
Consume two or more serving of fruits or vegetables per day	No Yes	89(13.7) 562(86.3)
Eat junk food regularly	No Yes	195(30.0) 456(70.0)
Keep too much salt in your diet	No Yes	341(52.4) 310(47.6)
Feel bad about yourself	No Yes	161(24.7) 490(75.3)
Feel no interest or pleasure in doing any things	No Yes	265(40.7) 386(59.3)
Feel hopeless	No Yes	131(20.1) 520(79.9)
Have smoking habit	No Yes	309(47.5) 342(52.5)
Have the habit of drinking alcohol	No Yes	609(93.5) 42(6.5)
Have blood pressure	No Yes	335(51.5) 316(48.5)
Have the presence of high cholesterol level	No Yes	216(33.2) 435(66.8)
Have any family history of heart failure disease	No Yes	286(43.9) 365(56.1)
Have the presence of anemia	No Yes	413(63.4) 238(36.6)
Have any type of diabetes	No Yes	255(39.2) 396(60.8)
Have the presence of hypertension	No Yes	295(45.3) 356(54.7)
Have sleep apnea problem	No Yes	212(32.6) 439(67.4)
Have irregular heart rhythms	No Yes	360(55.3) 291(44.7)
Have coronary artery disease	No Yes	536(82.3) 115(17.7)
Have angina symptoms	No Yes	330(50.7) 321(49.3)
Have kidney, lungs or other major disease	No Yes	580(89.1) 71(10.9)
Take statin to decrease cholesterol level	No Yes	429(65.9) 222(34.1)
BMI	Under weight Normal weight Overweight	37(5.7) 427(65.6) 187(28.7)

### Statistical analysis

**Fig. 1:**
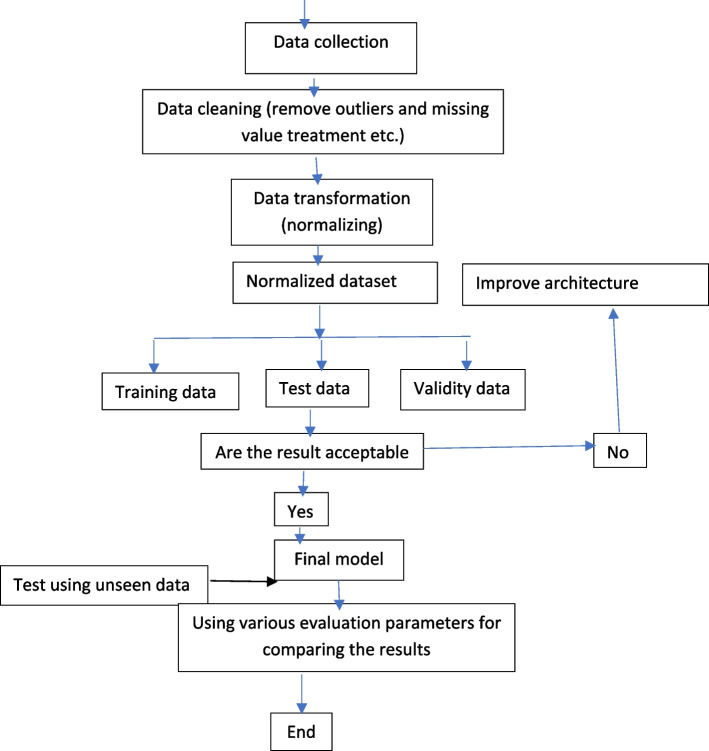
Workflow of the cardiovascular disease prediction model

Crosstabs were used to find descriptive statistics for both heart disease and the explanatory variables. The chi-square test was used to determine the association between heart disease and independent components. The features that contributed substantially were selected for machine learning (ML) training and categorization. A Python machine-learning classifier with fivefold cross-validation was used for the categorization. The classifiers used in this application include logistic regression, Naïve Bayes classifier, Decision Tree, AdaBoost classifier, Random Forest, Bagging Tree and Ensemble learning. The data were divided into test (20%) and training (80%) data sets (Fig. 1). The machine learning classifier's performance indicators were the area under the receiver operator characteristic (AU-ROC) curve, sensitivity, specificity, and accuracy. For statistical analysis, Python software was used at a 5% significance level.

### Different ML Techniques

#### Logistic regression

Machine learning techniques such as logistic regression, which are used to solve classification problems, are based on the concept of probability. When the target was categorical, it was used. This model converts probability to odds before calculating the logarithm of odds. The mathematical form of this model is,

$${\text{log}}\left[\frac{{P}_{i}}{1-{P}_{i}}\right]={\beta }_{0}+{\beta }_{1}{X}_{i1}+{\beta }_{2}{X}_{i2}+\dots +{\beta }_{k}{\beta X}_{ik}$$

where P_i_ denotes the probability of an event occurring and (1-P_i_) does not occur.

The ratio of the two represents the odds of an event. The left-hand side expresses the log-odds. ${\beta }_{0}$ is the intercept, which represents the mean value of log-odds when all independent variables are replaced by zero. ${\beta }_{1}$, ${\beta }_{2}$,…, ${\beta }_{k}$ are the coefficients of regression, measure the rate of change of log-odds due to change of independent variables (${X}_{i1},{X}_{i2},\dots {X}_{ik})$ [34].

It converts any real value to a range from zero to one using a sigmoid function. The sigmoid function appears as an S-shaped curve and can be defined as

$$f\left(x\right)=\frac{1}{1+{e}^{-x}}$$

However, a cost function, such as the cross-entropy loss, works in this regression system to measure the loss between the predicted probabilities and actual labels. The purpose of logistic regression is to minimize the cost function during the training phase [35]. Optimizing the hyperparameters is key to achieving the optimal performance of this algorithm. Machine-learning algorithms inherently rely on default parameter values if they are not manually adjusted by the user. For our primary dataset, we configured certain hyperparameters to tailor the behavior of the model. For instance, setting the "penalty = L2" dictates the norm used in penalization, while "C = 1.0" signifies the inverse of regularization strength. Additionally, "solver = lbfgs" specifies the optimization problem-solving approach. Other default parameters include "tol" (tolerance for stopping), "fit_intercept" (specifies whether to add a constant), "class_weight" (adjusts for class imbalance), "random_state" (random number generator for data shuffling), "max_iter" (maximum number of iterations), among others.

#### Naive bayes classifier

Naive Bayes is a supervised learning method that solves classification issues by applying the conditional probability concept of Bayes’ theorem. It is mostly employed for text categorization with a large training set. The underlying assumption is that the attributes have no correlation and are not connected to one another. Bayes’ theorem is written according to the following classification issue:

$$P\left(y|X\right)=\frac{P\left(X|y\right)P(y)}{P(X)}$$

where.

y = Targeted variable.

X = (x_1_,x_2_,x_3_,……,x_n_) = The input features.

P(y) = The prior knowledge about targeted variable.

P(X|y) = The likelihood functions.

When we substitute X and extending using the chain rule the Bayes theorem will be [36]

$$P\left(y|x1,x2,\dots xn\right)$$ α $$P(y) \prod_{i=1}^{n}P\left(X|y\right)$$

The model utilizes two parameters: "priors" for specifying the prior probabilities of the classes (set to none), and "var_smoothing" for incorporating variances to enhance stability (set to 1e-9).

#### Decision tree

Decision trees are supervised learning techniques that can be used to solve regression and classification problems; however, they are mostly employed to solve classification problems. It is a tree-structure classifier with two nodes for classifying unknown data. The decision nodes, which contain several branches, are utilized to make any decision, and the leaf nodes present the outcomes of these decisions. Attribute selection measures (ASM), such as information gain and selecting the best attribute for the root node and sub-node, are frequently achieved by employing the Gini index. Based on the information gain estimate, which provides us with how much information a feature informs us about a class, we divide the node and build the decision tree. An attribute with high information gain should be preferred as compared to low information gain and can be written as,

Information gain = Entropy(S)- [(Weighted Average) *Entropy (Each feature)].

Entropy = -P(yes) log2 P(yes)—P(no) log2 P(no).

Where, S = Total number of samples.

P(yes) = probability of yes.

P(no) = probability of no.

On the other hand, A measure of purity or impurity utilized by the classification and regression process to create a decision tree is the Gini index. A low Gini index should be chosen over a high Gini index. and can be calculated as,

Gini Index = 1- $$\sum_{j}{{P}^{2}}_{j}$$

P_j_ denotes the proportion of instances in which nodes correspond to class j [37].

The model's learning parameters include the following: criterion: defines the function used to assess split quality, splitter: determines the strategy for selecting splits at each node, max_depth: specifies the maximum depth of the tree, min_samples_split: sets the minimum number of samples required to split an internal node, min_samples_leaf: establishes the minimum number of samples required to form a leaf node, and min_weight_fraction_leaf: determines the minimum weighted fraction of the sum total of weights, max_features: specifies the number of features to consider when making splits, and random_state: ensures reproducibility by initializing the random number generator. The values assigned to these parameters are listed in Table 2.

**Table 2:** The values of parameters of some ML Models

Parameters	Used Parameters’ Values of Employed ML Models			
	**Decision Tree**	**Random Forest**	**Adaboost**	**Bagging**
Criterion	gini	gini		
Splitter	best			
Max_depth	none	None	1	None
Min_samples_split	2	2		
Min_samples_leaf	1	1		
Min_weigth_fraction_leaf	0.0	Sum total of weights		
Max_features	None	None		1.0
Random_state	x	None	None	42
N_estimators		100	50	10
Base_estimators			DecisionTree	RandomForest
Max_samples				1.0
Oob_score		false		false
bootstrap		true		true
N_jobs		none		none
Algorithm			SAMME	
Learning Rate			1	

#### AdaBoost classifier

The AdaBoost algorithm, which is also known as Adaptive Boosting, was proposed by Freund and Shapira. This is a machine learning ensemble method that uses boosting techniques for the final classification. It g generates n decision trees in the data-learning stage. When the decision tree is constructed, the incorrectly classified record from the original model is prioritized. Only these records were considered as the inputs for the second model. This process is repeated until we determine the number of basic learners that we want to generate. Recall that using all boosting strategies is acceptable for recording repetitions [38]. The tuning parameters that are used in this model for learning are Max_depth, Base_estimators: Represents the base estimator utilized to build the boosted ensemble.

Algorithm: Defines the algorithm employed to compute the weights for each classifier; learning _rate: Modifies the contribution of each classifier by shrinking it; N_estimators: Set the maximum number of estimators, indicating when boosting terminates. and Random_state. The values of these parameters are listed in Table 2.

#### Random forest

The Random Forest classifier is based on the principle of ensemble learning, which is the process of merging numerous classifiers to solve a complicated problem and enhance the model's performance. It employs a variety of decision trees on different subsets of the provided information and averages their results to increase the prediction accuracy of that dataset. Instead, depending on a single decision tree, the random forest collects forecasts from each tree and predicts the final output based on the majority vote of the predictions. The larger the number of trees in the forest, the higher the accuracy and lower the risk of overfitting. There are two phases in its operation: first, it builds a random forest by combining N decision trees, and then it predicts each tree that was built in the first stage. An attribute is selected using the information gain or Gini index for each decision tree [39]. The parameters used in this algorithm for learning are Criterion, Max_depth, Min_samples_split, Min_samples_leaf, Min_weigth_fraction_leaf, Max_features (the number of features to draw from X to train each base estimator), N_estimators, Random_state, oob_score (whether to use out-of-bag samples to estimate the generalization accuracy), bootstrap (whether bootstrap samples are used when building trees), and N jobs (the number of jobs to run in parallel for both fit and predict). Table 2 lists the values of the tuning parameters.

#### Bagging tree

Bagging, also referred to as bootstrap aggregating, is an ensemble learning method that enhances the efficiency and precision of machine-learning algorithms. It uses a bootstrapping approach to create random samples of data from a population and estimates a population parameter. We assume that the training set consists of n observations and m features. Next, a random sample was selected from the training dataset without replacement. A random subset of m characteristics was chosen to create a model using sample data. The attribute that yields the optimal split among all nodes is used to divide them. Because the tree was completely formed, we had the largest number of root nodes. The above-listed processes are completed ‘n’ times. It integrates the output from each individual decision tree to produce the most accurate forecast. The integrated classifier prediction is a weighted aggregate of separate classifier predictions and can be written as

$$H\left(di\right)=sign(\sum_{m=1}^{M}{\alpha }_{m}{H}_{m}(di)$$

where, $H\left(di\right)=$ For a given instance di, this is the ultimate decision function. This is the result of weighting the various classifiers by their respective coefficients.

Sign(.) = This function accepts the argument's sign and returns + 1 in the case of a positive argument, -1 in the case of a negative argument, and 0 in the case of a zero argument. This is used to determine a final conclusion in binary classification by considering the sign of the weighted sum.

M is the total number of classifiers in the ensemble.

Α represents the weight and ${H}_{m}\left(di\right)=$ For the instance di, this is the prediction of the m^th^ classifier [40]. The parameters used in this model for learning are Max_depth, Max_features, Max_samples (meaning it uses all(1) samples or not(0)), Base_estimators, N_estimators, Random_state, oob_score, bootstrap, and N-jobs. Table 2 lists the values of the parameters used in this algorithm.

#### Ensemble learning techniques

**Fig. 2:**
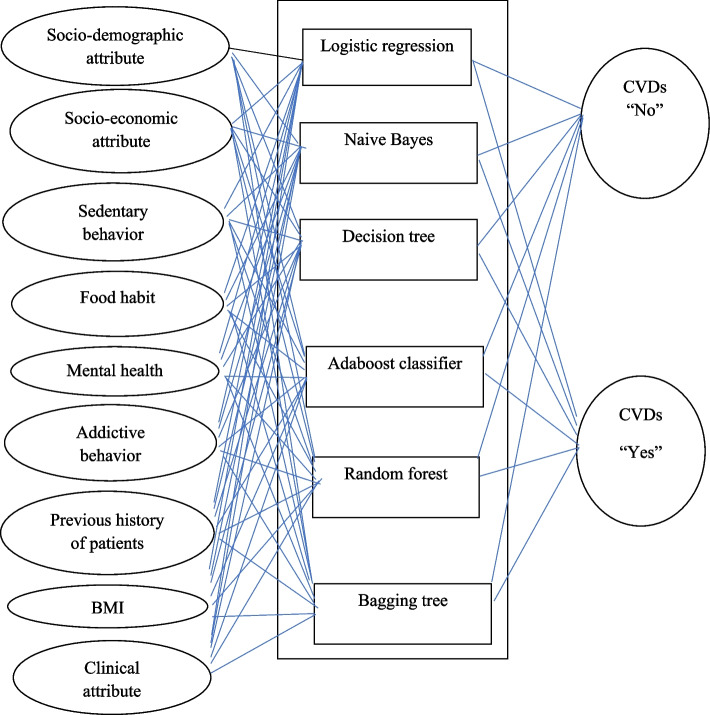
Cardiac failure prediction model structure

Ensemble learning is a strategy that integrates many machine-learning algorithms to generate a single optimum predictive model with decreased volatility (by bagging), bias (via boosting), and enhanced predictions (via stacking). This method offers robustness against data uncertainties and improves accuracy. Boosting, stacking, and bagging are the three primary categories of ensemble learning techniques [41] (Fig. 2).

## Results

### Descriptive statistics

The mean age of the respondents was 57.21 years. Among them, 60% were male and 37% were female (Table 1). Approximately 60.1% of the participants in the sample had cardiovascular disease, whereas the remaining 39.9% were not affected by any type of cardiac failure. The dataset contains several medical disorders, including high cholesterol (66.8%), hypertension (54.7%), and diabetes (60.8%). Most participants (65.6%) were normal weight, 28.7% were overweight, and 5.7% were underweight. Average Platelet's level, creatinine level, and sodium level are 263,430.47 mcl (150,000–400000) mcl, 1.777 mg\dl(0.40–1.40 mg\dl), and 146.335 mmol\L (135–148 mmol\L), respectively (Table 3).

**Table 3:** Summary statistics (continuous) of different variables of Cardiovascular patient

Variable	Mean	Minimum	Maximum	Standard deviation
Age	57.21	22	89	15.145
Platelet's level	263,430.47	29,000	476,000	47,227.831
Creatinine level	1.777	0.6	6.8	0.9369
Sodium level	146.335	0.8	234.0	15.2348

Primary education constituted the highest percentage of the sample (38.4%). Moreover, 17.1% had no education, 26.6% had a secondary education, and 18.0% had a higher secondary education. Most participants (58.8%) came from a middle-income family (20,000–40,000), whereas the remaining 21.4% had low-income (< 20,000), and 19.8% belonged to high-income (> 40,000) families. Most participants lived in rural areas (61%). According to the table, 67.3% of respondents engaged in regular physical activity. Approximately 86.3% of the population consumes two or more servings of fruits or vegetables each day, and 70.0% do not consume junk food on a regular basis. More than half (55.9%) of the participants slept at night (Table 1).

A significant proportion of the respondents reported different negative mental health indicators, such as feeling bad about themselves (75.3%), feeling hopeless (79.9%), and having little interest or pleasure in doing activities (59.3%). Among the participants, 47.5% smoked, and 6.5% drank alcohol.

According to the chi-square test, there was a significant correlation between gender, respondents' educational levels, socio-economic status, regular physical exercise, sound sleep at night, eating junk food regularly, keeping too much salt in your diet, feeling bad about yourself, feeling hopeless, having a smoking habit, having a habit of drinking alcohol, having blood pressure, having a high cholesterol level, having any family history of heart failure disease, having anemia, having any type of diabetes, having hypertension, having a sleep apnea problem, having irregular heart rhythms, coronary artery disease, angina symptoms, kidney, lung, or other major diseases, BMI, and CVD. The chi-square test results suggested a significant correlation between numerous variables and the presence of CVD, all of which had a p-value of less than 0.05. However, there was no discernible link between CVD and division, residence, consuming two or more servings of fruits or vegetables daily, and feeling no interest or pleasure in doing anything (Table 4).

**Table 4:** Relationship between different variables and cardiovascular disease

Variables	Category	Have you CVD?		*P* value
		NO (n)	YES (n)	
**Gender**	Female	110	131	0.023
	Male	150	260	
**Division**	Dhaka	34	94	0.111
	Chattogram	60	82	
	Khulna	29	30	
	Rajshahi	20	34	
	Barisal	34	35	
	Sylhet	29	31	
	Mymenshing	21	30	
	Rangpur	33	55	
**Education**	No education	27	84	0.000
	Primary	98	152	
	Secondary	64	109	
	Higher secondary	71	46	
**Socio-economic status**	< 20,000	41	98	0.000
	20,000–40,000	140	243	
	> 40,000	79	50	
**Residence**	Urban	99	155	0.688
	Rural	161	236	
**Take physical exercise regularly**	No	103	110	0.002
	Yes	157	281	
**Have sound sleep at night**	No	50	237	0.000
	Yes	210	154	
**Consume two or more serving of fruits or vegetables per day**	No	36	53	0.502
	Yes	224	338	
**Eat junk food regularly**	No	124	71	0.000
	Yes	136	320	
**Keep too much salt in your diet**	No	182	159	0.000
	Yes	78	232	
**Feel bad about yourself**	No	151	10	0.000
	Yes	109	381	
**Feel no interest or pleasure in doing any things**	No	113	152	0.243
	Yes	147	239	
**Feel hopeless**	No	110	21	0.000
	Yes	150	370	
**Have smoking habit**	No	186	123	0.000
	Yes	74	268	
**Have the habit of drinking alcohol**	No	235	374	0.006
	Yes	25	17	
**Have blood pressure**	No	220	115	0.000
	Yes	40	276	
**Have the presence of high cholesterol level**	No	199	17	0.000
	Yes	61	374	
**Have any family history of heart failure disease**	No	194	92	0.000
	Yes	66	299	
**Have the presence of anemia**	No	228	185	0.000
	Yes	32	206	
**Have any type of diabetes**	No	175	80	0.000
	Yes	85	311	
**Have the presence of hypertension**	No	232	63	0.000
	Yes	28	328	
**Have sleep apnea problem**	No	192	20	0.000
	Yes	68	371	
**Have irregular heart rhythms**	No	242	118	0.000
	Yes	18	273	
**Have coronary artery disease**	No	250	286	0.000
	Yes	10	105	
**Have angina symptoms**	No	229	101	0.000
	Yes	31	290	
**Have kidney, lungs or other major disease**	No	252	328	0.000
	Yes	08	63	
**BMI**	Under weight	11	26	0.001
	Normal	193	234	
	Overweight	56	131	
**Take statin to decrease cholesterol level**	No	230	199	0.000
	Yes	30	192	

### Implementation and analysis of different machine learning models

This study employed multiple ML models to predict CVDs in Bangladesh. The effectiveness of the employed ML models was analyzed by determining the confusion matrix, and a comparison among all employed ML techniques was also conducted. The next section examines the data and unveils its discoveries, paving the way for the subsequent section that delves into the assessment of performance across different classification techniques.

### Data analysis

**Fig. 3:**
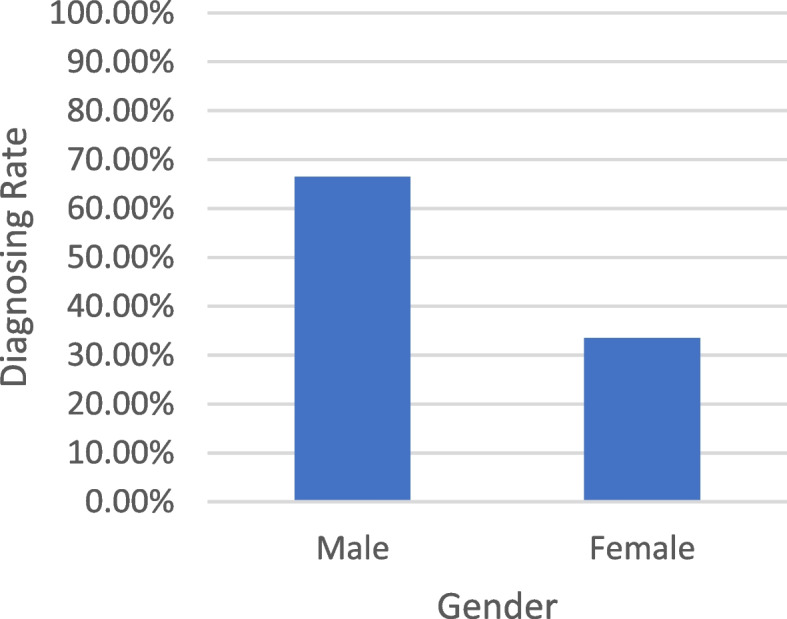
Relationship Between Gender features and CVD

The collected data were scrutinized and categorized into male and female segments, as illustrated in Fig. 3 and Table 5. Of a total of 651 samples, 391 individuals were diagnosed with CVD. The Analysis further indicated that the incidence rates in males and females were 66.5% and 33.5%, respectively. Notably, the mean number of males diagnosed with heart disease exceeded that of females.

**Table 5:** Analysis of CVD Dataset

*Category*		*No. of the diagnosed* *person*	*Diagnosing Rate*
*Gender*	Male	260	66.5%
	Female	131	33.5%
*Total*	Total Sample	651	
	Total Diagnosed Sample	391	

### Performance analysis

To assess and gauge the efficacy of the employed algorithms, a comprehensive evaluation was conducted using the confusion matrix and an array of pertinent metrics, which encompassed the ROC curve, True Positives, True Negatives, False Positives, False Negatives, precision, recall, F1 score, and accuracy. In the subsequent section, we present a performance analysis of each algorithm.

### Logistic regression

**Fig. 4:**
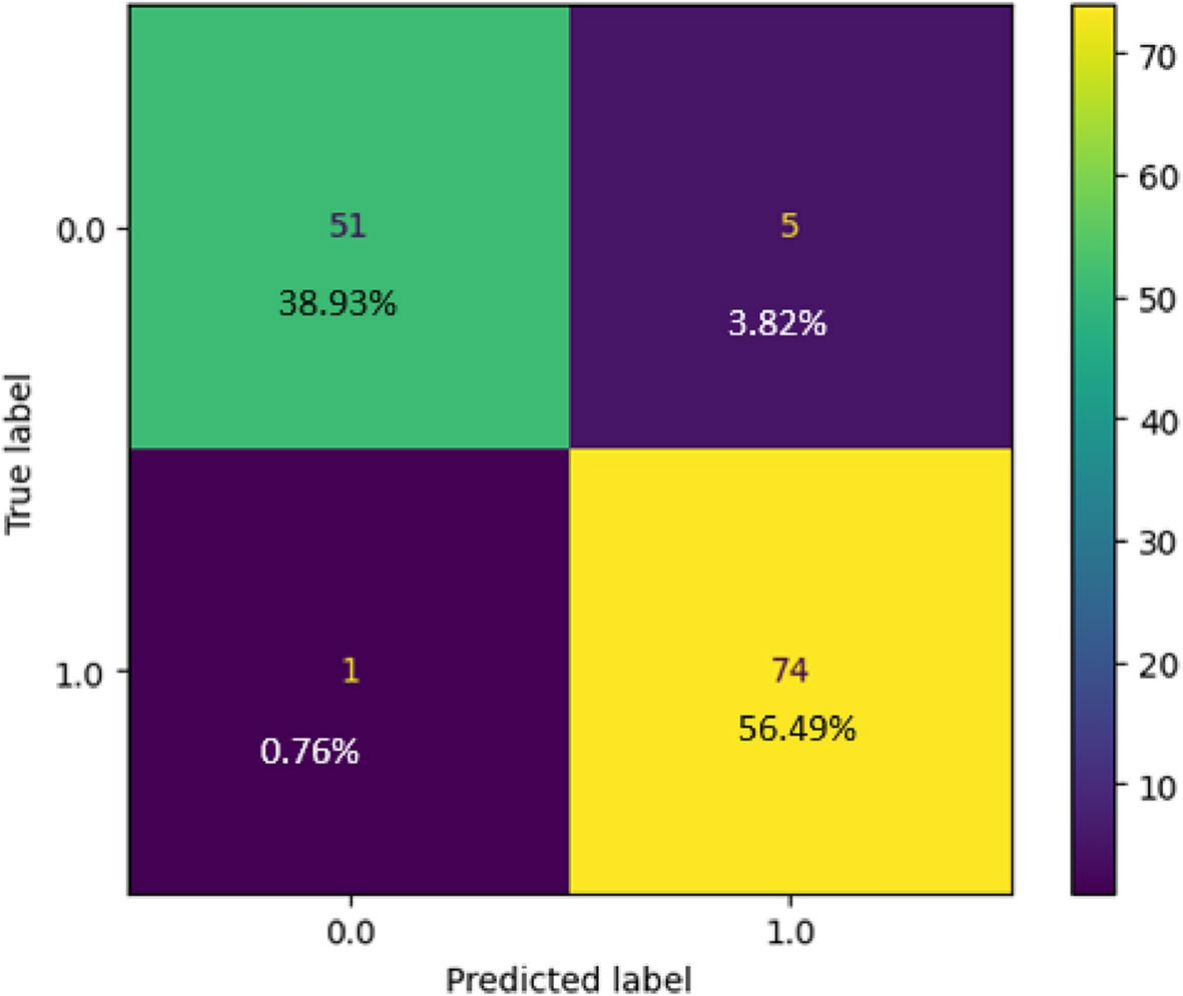
Confusion Matrix of Linear Regression

**Fig. 5:**
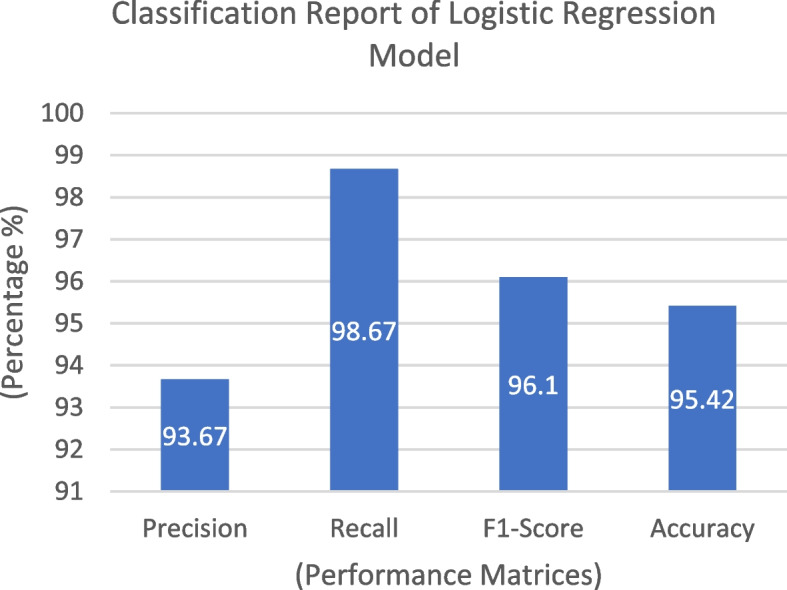
Classification Report of Linear Regression Model

The logistic regression method was developed on a dataset containing 520 samples and subsequently tested using 131 samples following the train-test split paradigm. Upon analyzing the performance of the model, we obtained the confusion matrix represented in Fig. 4. In this matrix, the yellow and green cells indicate correct predictions, where the model's output matches the target, whereas the purple cell signifies instances where there is a mismatch with the target. Figure 4 reveals that the Logistic Regression model accurately predicted 51 cases of no CVD and incorrectly predicted five samples. The model correctly identified 74 CVD cases. Consequently, the total number of correct predictions was 125, whereas there were six instances of incorrect predictions. As a result of this analysis, the model's overall accuracy was calculated as 95.42%, as depicted in Fig. 5. Additionally, the precision rate of the model was 93.67%, and the recall rate was 98.67%. Upon examining the F1 score (96.1%) in Fig. 5, it is evident that the model strikes a commendable balance between achieving precise positive predictions and correctly capturing the most positive instances.

**Fig. 6:**
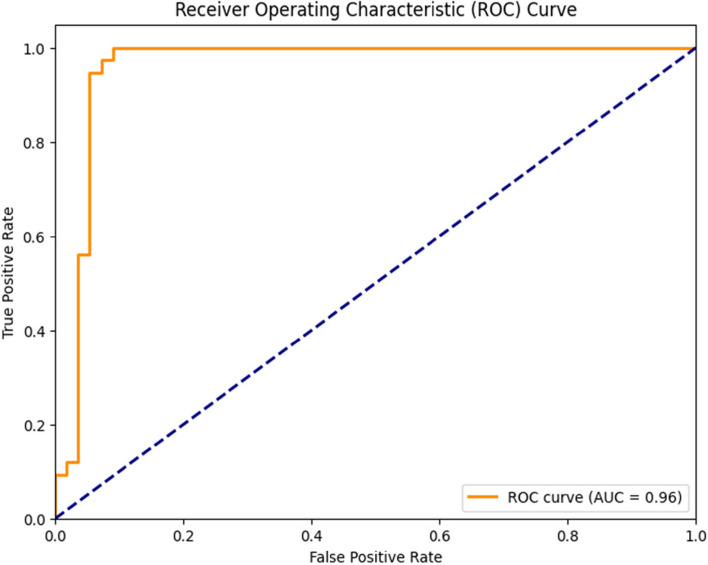
ROC Curve of Linear Regression

Figure 6, on the other hand, represents the ROC (Receiver Operating Characteristic) curve for the Logistic Regression model. In this representation, the Y-axis corresponds to the true positive rate, whereas the X-axis represents the False Positive Rate. Notably, the Area Under the ROC Curve (AUC) was calculated as 0.96 for both classes, signifying a high level of discriminative power and effectiveness in distinguishing between classes.

### Naïve bayes classifier

**Fig. 7:**
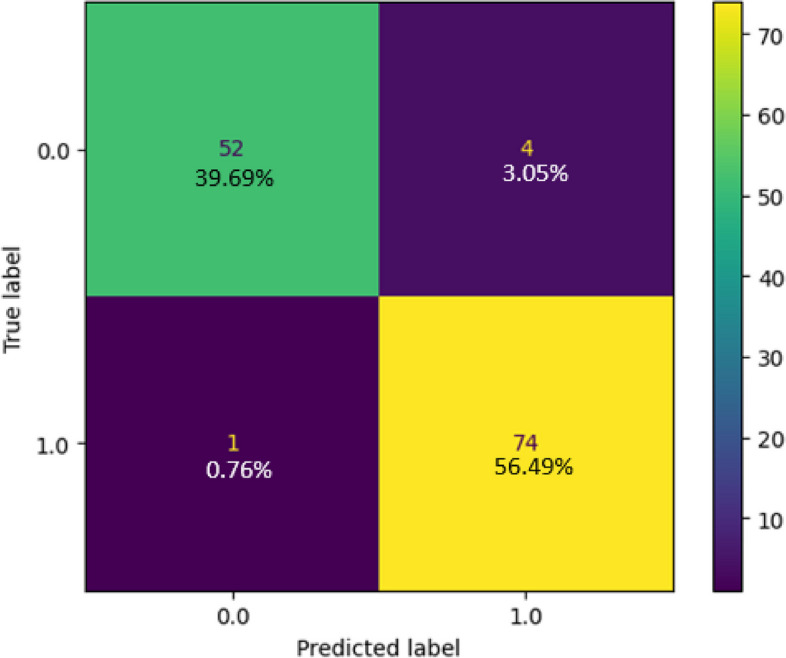
Confusion Matrix of Naïve Bayes

**Fig. 8:**
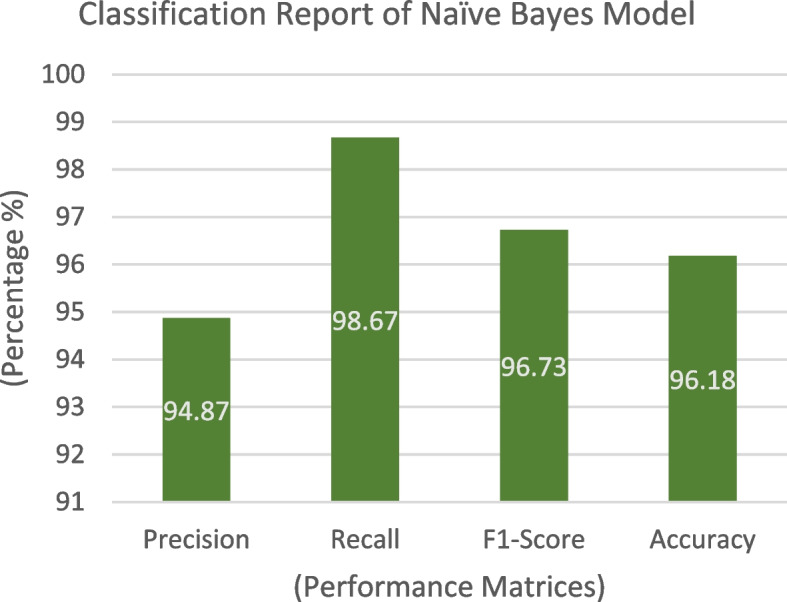
Classification Report of Naïve Bayes Model

**Fig. 9:**
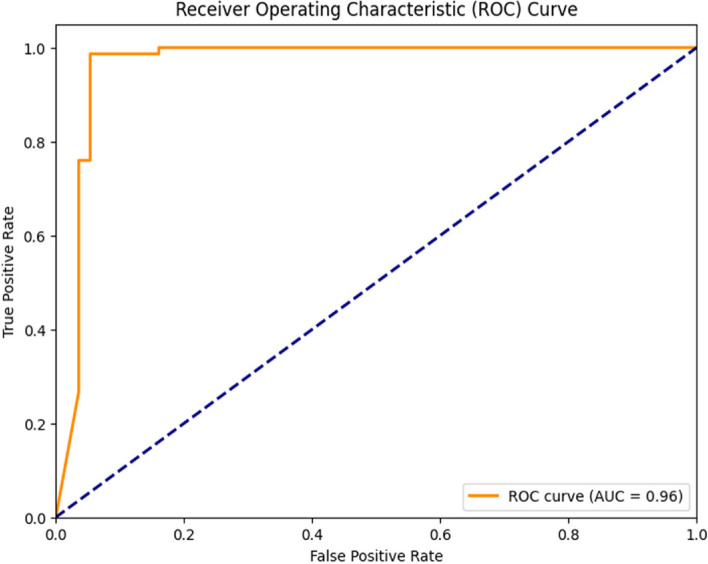
ROC Curve of Naïve Bayes

The confusion matrix derived from testing the Naïve Bayes model on the collected dataset is illustrated in Fig. 7. This matrix presents the predictions of the model in relation to test data. Of the 131 test samples, the classifier accurately predicted 74 samples for Class 1 and 52 samples for Class 0. Furthermore, there was one incorrect prediction for the positive class and four incorrect predictions for the negative class. The number of correct predictions was 126 with five instances of incorrect predictions. The classification report of the technique is provided in Fig. 8, where we can ascertain that the model achieved an accuracy of 96.18%, while the error rate was 3.82%. The model excelled in positive predictions, with a robust precision rate of 94.87% and an impressive recall of 98.67%. This high precision minimizes false positives, while strong recall captures an important portion of actual positive cases, showcasing the model's proficiency. With an F1 score of 96.73%, the model maintains a fine balance between precision and recall, making accurate positive predictions while comprehensively capturing positive instances. Figure 9 displays the ROC curve, illustrating the model's performance with an AUC of 0.96 for both positive and negative classes, confirming its strong ability to distinguish between classes in binary classification tasks.

### Decision tree classifier

**Fig. 10:**
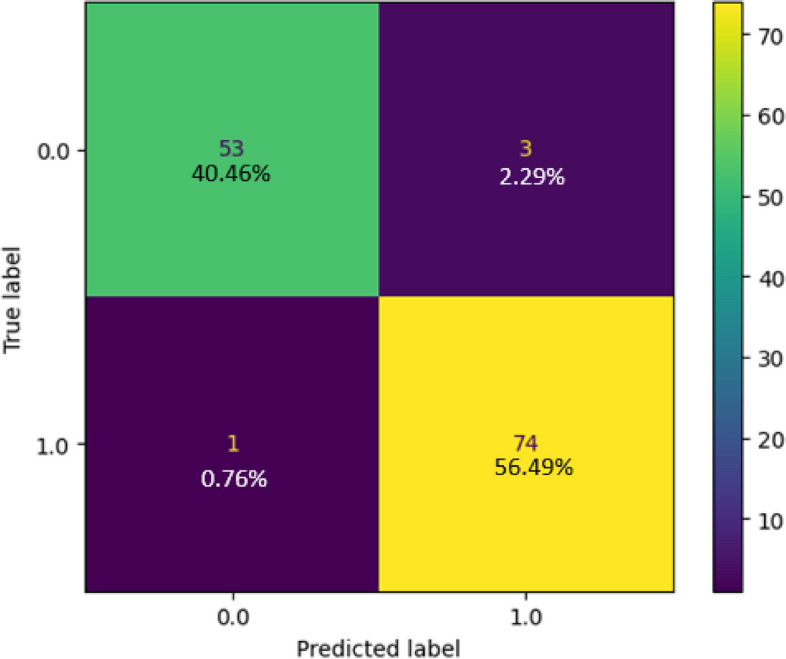
Confusion Matrix of Decision Tree Classifier

**Fig. 11:**
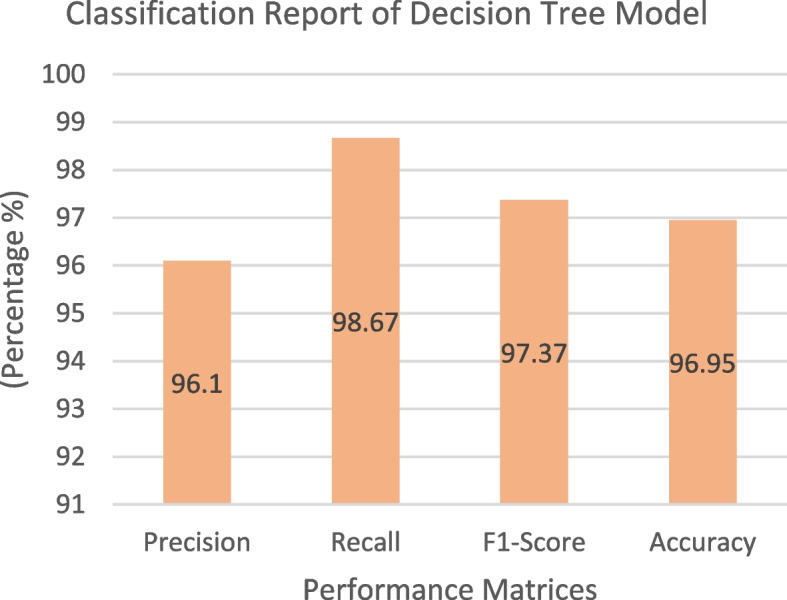
Classification Report of Decision Tree Classifier

**Fig. 12:**
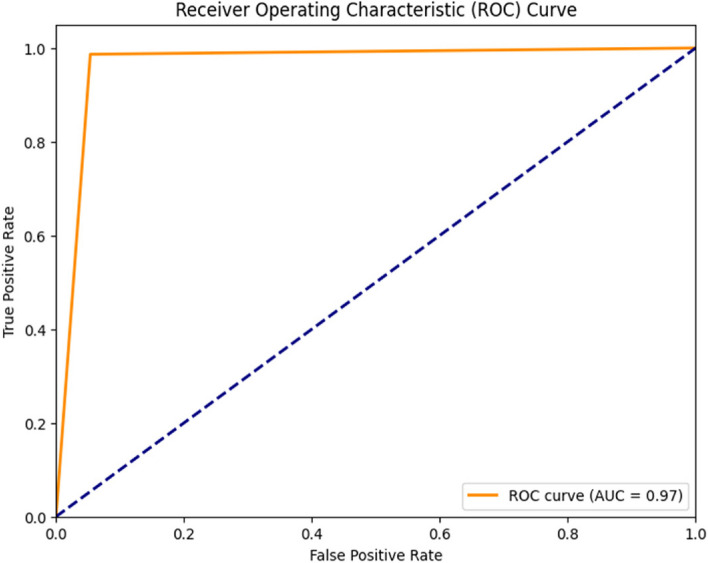
ROC Curve of Decision Tree Classifier

The collected dataset was used to train and test a Decision Tree classifier, and the resulting confusion matrix is displayed in Fig. 10. In this matrix, the green and yellow cells indicate that the model's output class matches the target class, whereas the purple cell signifies instances in which the model's output class does not align with the target class. For Class 1, the classifier correctly predicted 56.49% (74) of the samples and made incorrect predictions in only 0.76% (1) of the cases. For Class 0, the classifier accurately predicted 40.46% (53) of the samples and had only 2.29% (3) incorrect predictions. The Decision Tree classifier correctly identified 127 instances and had four instances with incorrect predictions out of 131 samples. As shown in Fig. 11, the classification report highlights the performance of the model. It exhibited a notably high true-positive rate of 98.67%, reflecting its ability to effectively capture positive instances. Furthermore, the precision rate was commendable at 96.1%. This balance between correct and incorrect predictions (97.37%) underscores the model's overall acceptability and effectiveness. Figure 12 shows the ROC curve of the classifier. Impressively, the Area Under the ROC Curve (AUC) measures 0.97 for both classes 0 and 1, indicating a high level of discriminatory power and effectiveness in distinguishing between the two classes.

### AdaBoost classifier

**Fig. 13:**
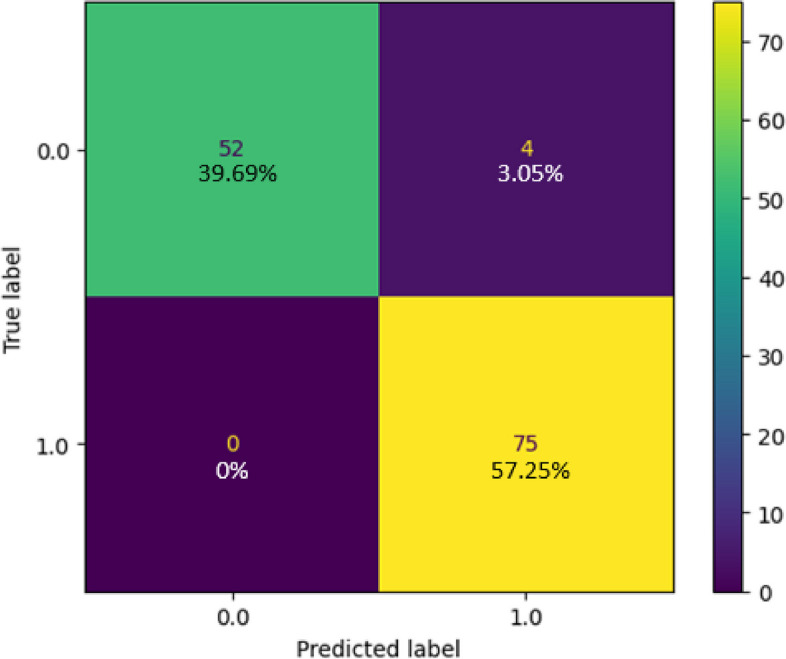
Confusion Matrix of AdaBoost Classifier

**Fig. 14:**
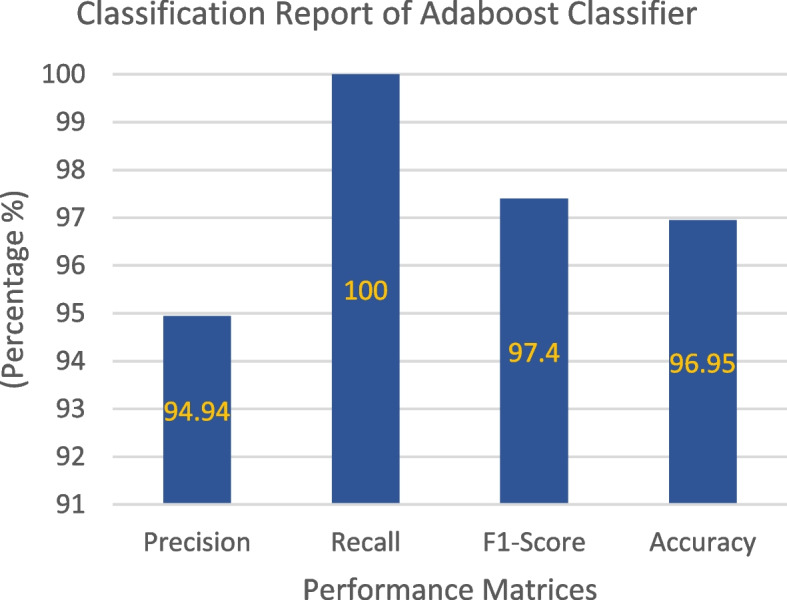
Classification Report of AdaBoost Classifier

**Fig. 15:**
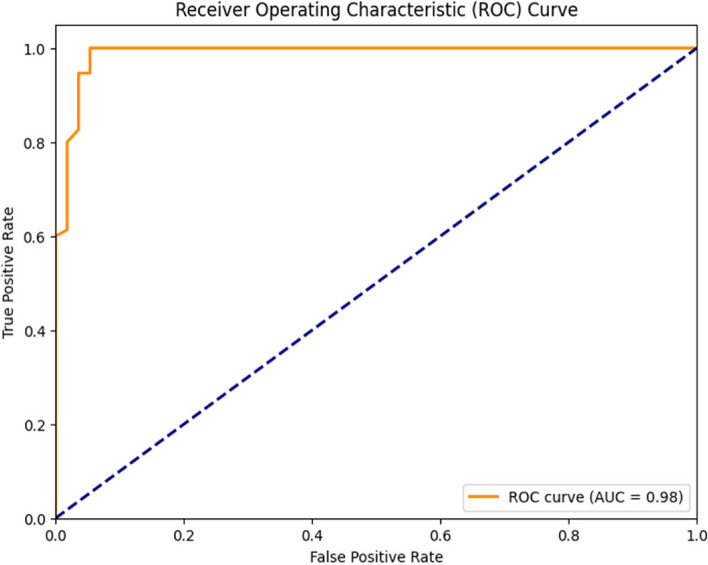
ROC Curve of AdaBoost Classifier

The confusion matrix generated by testing the AdaBoost Classifier on the collected dataset is shown in Fig. 13. This matrix represents the model predictions of the test data. Of the 131 test samples, the classifier correctly predicted 75 samples, accounting for 57.25% of the total, for Class 1, and 52 samples, which corresponded to 39.69% for Class 0. Notably, the model did not make any incorrect predictions for class 1, and only four (3.05%) incorrect predictions for class 0. The total instances of accurate and erroneous predictions totaled 127 and four, respectively. The classification report of the model is presented in Fig. 14, revealing that the model achieved a remarkable accuracy of 96.95%, with an error rate of only 3.05%. The model excelled in making positive predictions, boasting an impressive precision rate of 94.94% and a perfect recall of 100%. With an F1 score of 97.4%, the classifier's predictions exhibited an exceptional balance between precision and recall, underlining its proficiency. Figure 15 presents the ROC curve, depicting the false-positive rate on the x-axis and the true-positive rate on the y-axis. Impressively, the Area Under the ROC Curve (AUC) measures 0.98 for both positive and negative classes, affirming the model's strong discriminatory power and effectiveness in distinguishing between the two classes in binary classification tasks.

### Random forest classifier

**Fig. 16:**
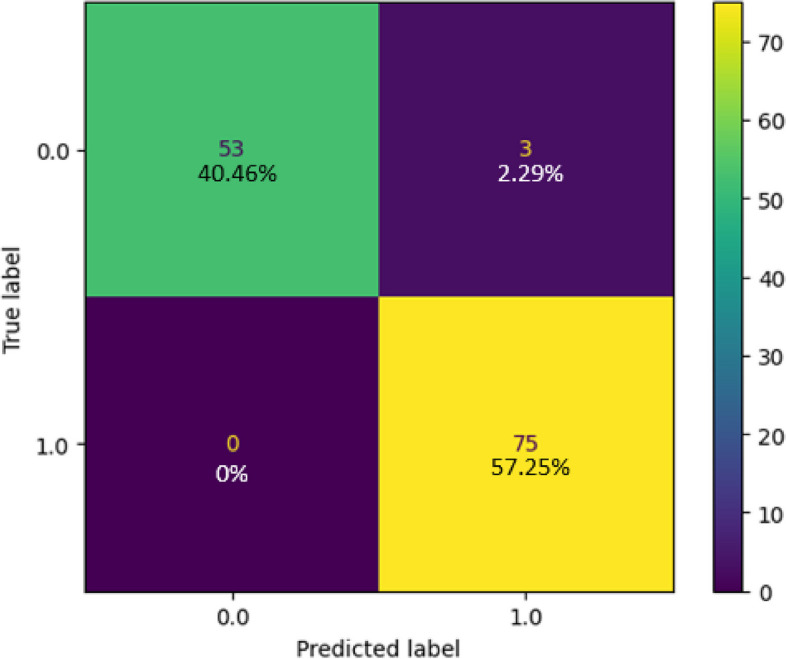
Confusion Matrix of Random Forest Classifier

**Fig. 17:**
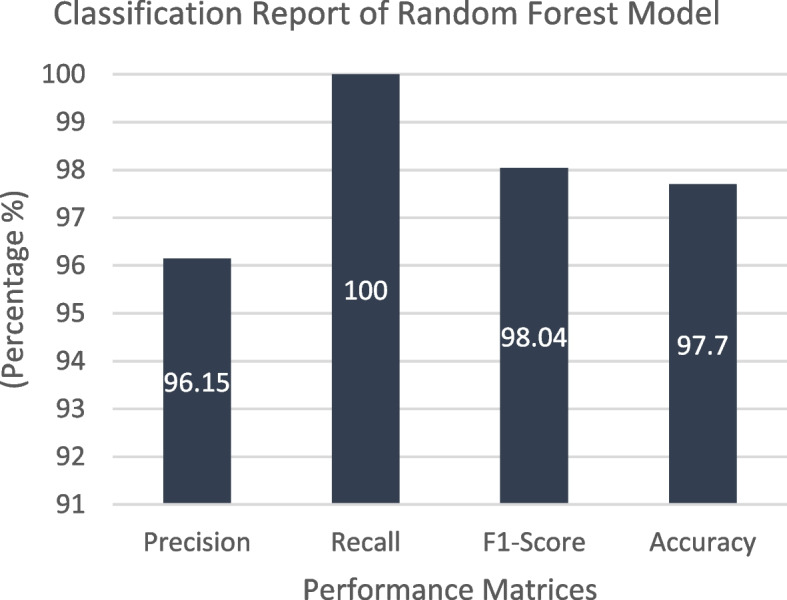
Classification Report of Random Forest Classifier

**Fig. 18:**
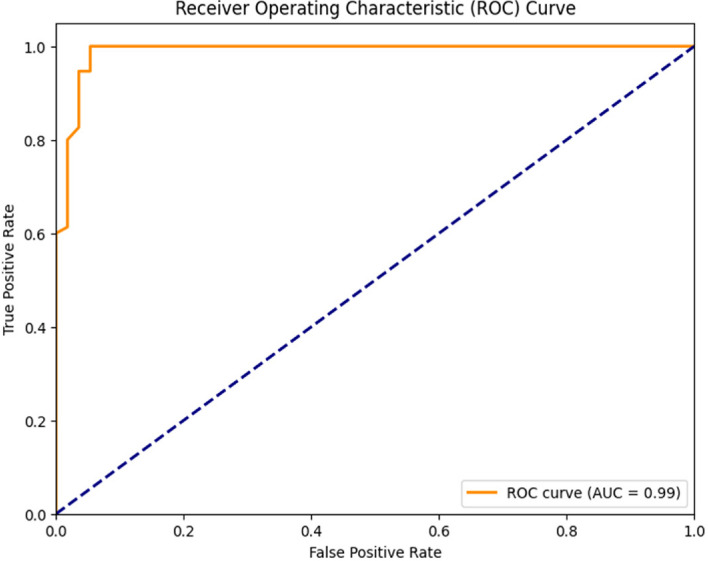
ROC Curve of Random Forest Classifier

The collected dataset served as the basis for training and testing a Random Forest classifier, and the resulting confusion matrix is depicted in Fig. 16. In this matrix, the green and yellow cells indicate instances where the model’s resulting class corresponds to the goal class, while the purple cell signifies cases where the model's outcome class does not match the target class. For Class 1, the classifier made correct predictions for all 75 samples, achieving a 57.25% accuracy rate. In the case of class 0, the classifier accurately predicted 40.46% (53) of the samples while making 2.29% (3) incorrect predictions. In total, the Random Forest classifier accurately identified 128 instances and had three instances with incorrect predictions out of 131, resulting in an impressive overall accuracy of 97.7%. Figure 17 presents the classification report of the classifier in use, demonstrating a perfect true-positive rate of 100% and a commendable precision rate of 96.15%. The balance between correct and incorrect predictions was notably high at 98.04%, signifying the model's very good acceptability. Figure 18 shows the ROC curve of the classifier, where the Area Under the ROC Curve (AUC) reaches an impressive 0.99 of 0 and class 1. This high AUC value underscores the classifier's exceptional ability to distinguish between two classes in binary classification tasks.

### Bagging tree

**Fig. 19:**
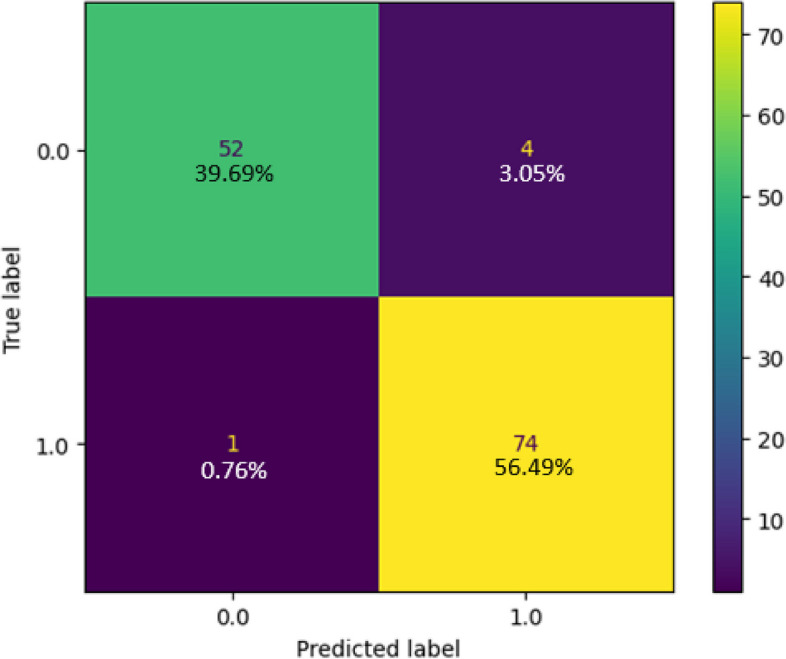
Confusion Matrix of Bagging Tree Classifier

**Fig. 20:**
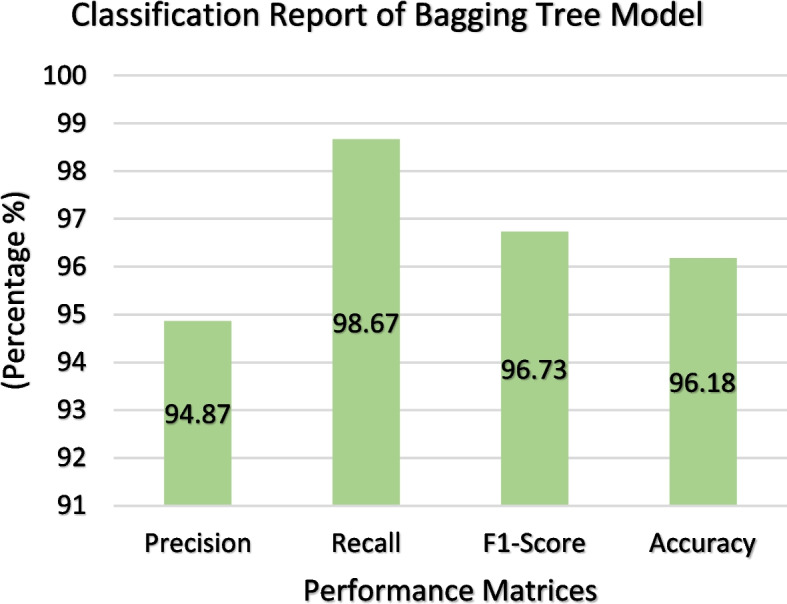
ROC Curve of Bagging Tree ClassifierClassification Report of Bagging Tree Classifier

**Fig. 21:**
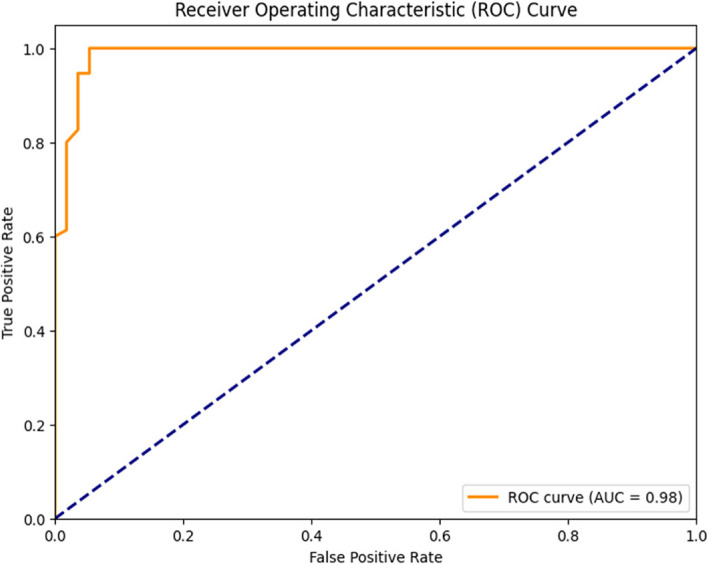
ROC Curve of Bagging Tree Classifier

The Bagging Tree model was trained on a dataset comprising 520 samples and subsequently tested using 131 samples following the train-test split methodology. After scrutinizing the performance of the tested model, we derived the confusion matrix shown in Fig. 19. In this matrix, the yellow and green cells signify instances where the output of the model aligns with the target, whereas the purple cell denotes cases where there is a mismatch. As shown in Fig. 19, the Bagged Tree model accurately predicts 52 samples, but it makes incorrect predictions for five samples in the context of heart disease. However, 74 instances of heart disease were correctly identified. The model achieved 126 correct predictions and seven incorrect predictions, resulting in an overall accuracy of 96.18%, as depicted in Fig. 20. In addition, the precision and recall rates were 94.87% and 98.67%, respectively. The F1 score, also shown in Fig. 19, indicates an excellent mix in producing precise positive forecasts and catching the majority of actual positive cases. Figure 21 presents the ROC curve of the Bagged Tree model, where the Y-axis denotes the True Positive Rate and the X-axis represents the False Positive Rate. Impressively, the Area Under the ROC Curve (AUC) measures 0.98 for both classes 1 and 0, signifying the model's strong ability to distinguish between the two classes effectively in binary classification tasks.

### Comparative analysis

**Fig. 22:**
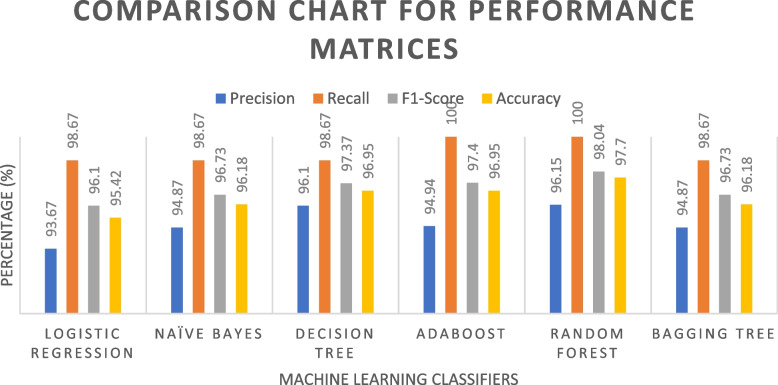
Comparison Chart for Performance Matrices among Employed Classifiers

**Fig. 23:**
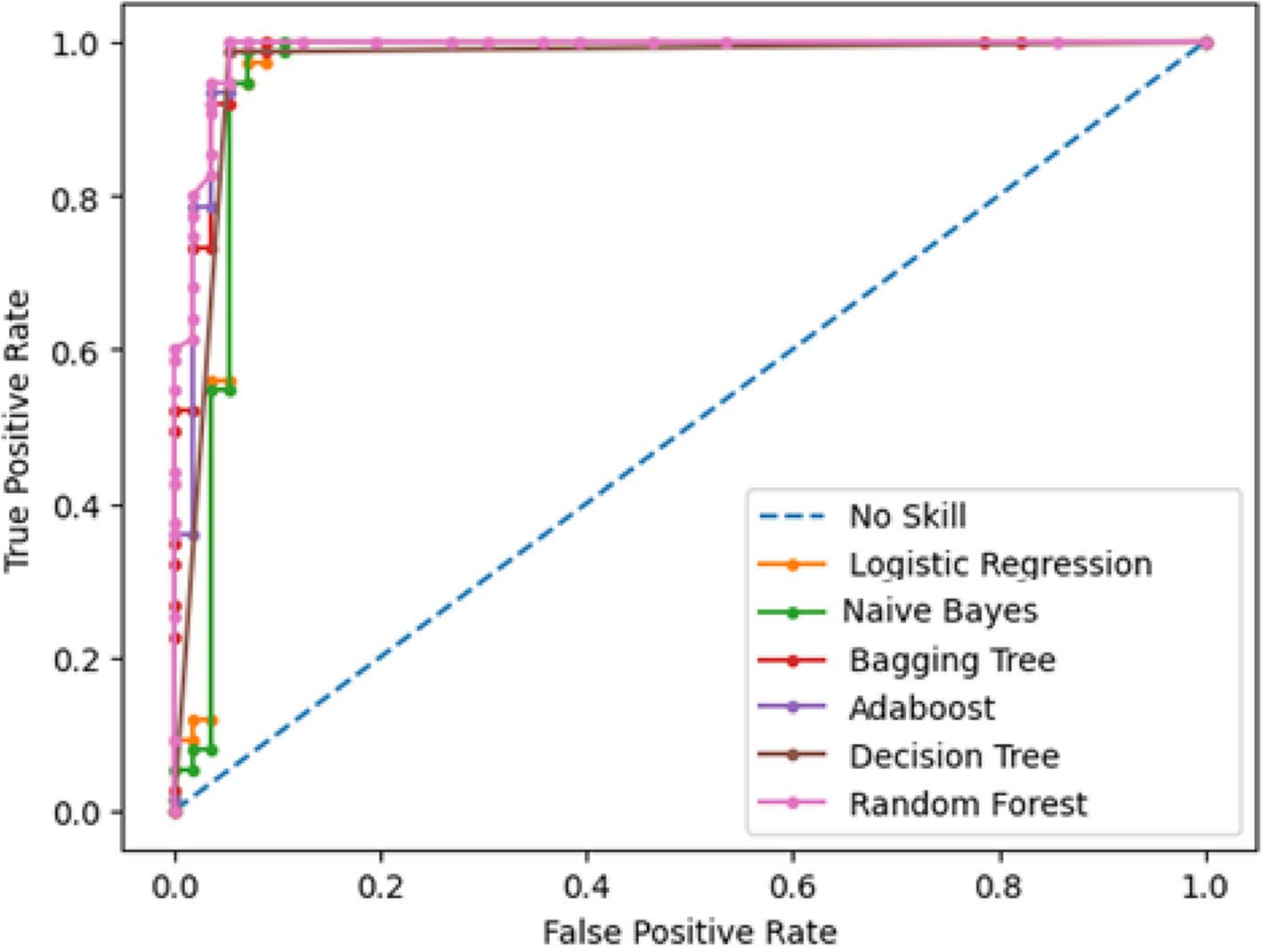
Comparison Graph of Area Under ROC curve

A comparative analysis was conducted among several classifiers: logistic regression, naïve Bayes, decision tree, AdaBoost, Random Forest, and bagging tree. This assessment thoroughly examined the performance metrics and ROC curves, as depicted in Figs. 22 and 23. The performance of these classifiers is further compared in Table 6, focusing on the precision, recall, F1 score, accuracy, and ROC. The precision rates for the mentioned classifiers are as follows: Logistic Regression (93.67%), Naïve Bayes (94.87%), Decision Tree (96.1%), AdaBoost (94.94%), Random Forest (96.15%), and Bagging Tree (94.87%). Among the five techniques considered, Random Forest stands out as having the highest precision. Furthermore, all classifiers demonstrated exceptional true positive rates, with both AdaBoost and Random Forest achieving a perfect 100% positive rate. The Random Forest classifier maintained the highest balance between correct and incorrect predictions, boasting an impressive rate of 97.7%. Although other models perform well, they do not match random forests in this regard. With its high precision, robust recall, and strong F1 score, the Random Forest classifier also achieved the highest accuracy of 98.04%. In contrast, the Logistic Regression model achieved the lowest accuracy, with clocking at 95.42%. Figure 23 and Table 6 provide clear evidence of the Area Under ROC Curve (AUC) for Class 0 and Class 1. Across the classifiers, the AUC values are as follows: Logistic Regression (0.959), Naïve Bayes (0.957), Decision Tree (0.967), AdaBoost (0.984), Random Forest (0.989), and Bagging Tree (0.985). Remarkably, the Random Forest classifier attained the highest AUC value, with an impressive value of 0.989.

**Table 6:** Comparison Table of Matrices among Different Classifiers

	**Logistic Regression**	**Naïve Bayes**	**Decision Tree**	**AdaBoost**	**Random Forest**	**Bagging Tree**
*Precision*	93.67%	94.87%	96.1%	94.94%	96.15%	94.87%
*Recall*	98.67%	98.67%	98.67%	100%	100%	98.67%
*F1-Score*	96.1%	96.73%	97.37%	97.4%	98.04%	96.73%
*Accuracy*	95.42%	96.18%	96.95%	96.95%	97.7%	96.18%
*ROC*	0.959	0.957	0.967	0.984	0.989	0.985

The selected classifiers are applied to assess entirely new samples that have not been previously tested. The algorithm's prediction process consists of the following steps:

i. Evaluation of the dataset with fresh cases.
ii. Following the learning phase, the entire model was delivered from the software application to the workspace for further prediction.
iii. Next, the new test dataset was uploaded, ensuring that it was appropriately normalized. This dataset should maintain identical attribute fields as the previous complete training dataset, with the sole exception of the lack of target class values.
iv. Within the working environment, define a dedicated function for each of the exported trained models, following the format 'yfit = trainedmodel.predictFunction(T)'. Here,” trained model ' corresponds to the name of the compact model, and 'T' is the reference to the test dataset.
v. Execute the evaluation of the test dataset, and subsequently employ various classifier algorithms for testing.

The primary objective of this research is to identify and employ an algorithm that outperforms the existing early prediction systems for heart diseases. In pursuit of this goal, we aim to enhance the accuracy of heart disease prediction. This research was motivated by the critical need to develop more effective and reliable methods for the early detection and prognosis of heart diseases. By exploring a range of machine learning and statistical modeling approaches, we aim to discover an algorithm that can greatly increase the predictability and efficiency of liver damage, ultimately contributing to better patient care and healthcare outcomes.

### Proposed classifier

**Fig. 24:**
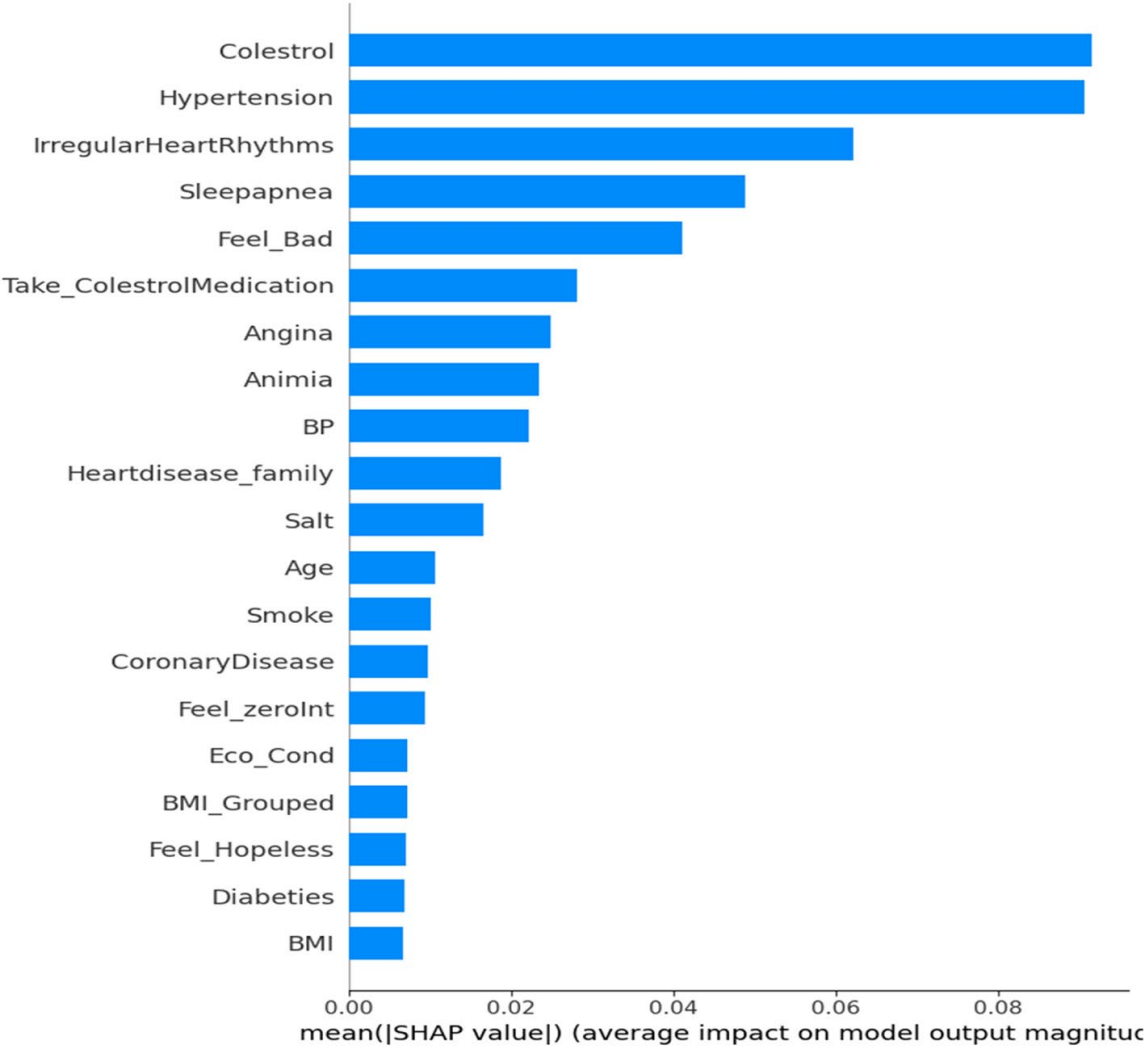
Average Impact of Each Features on Model Prediction

**Fig. 25:**
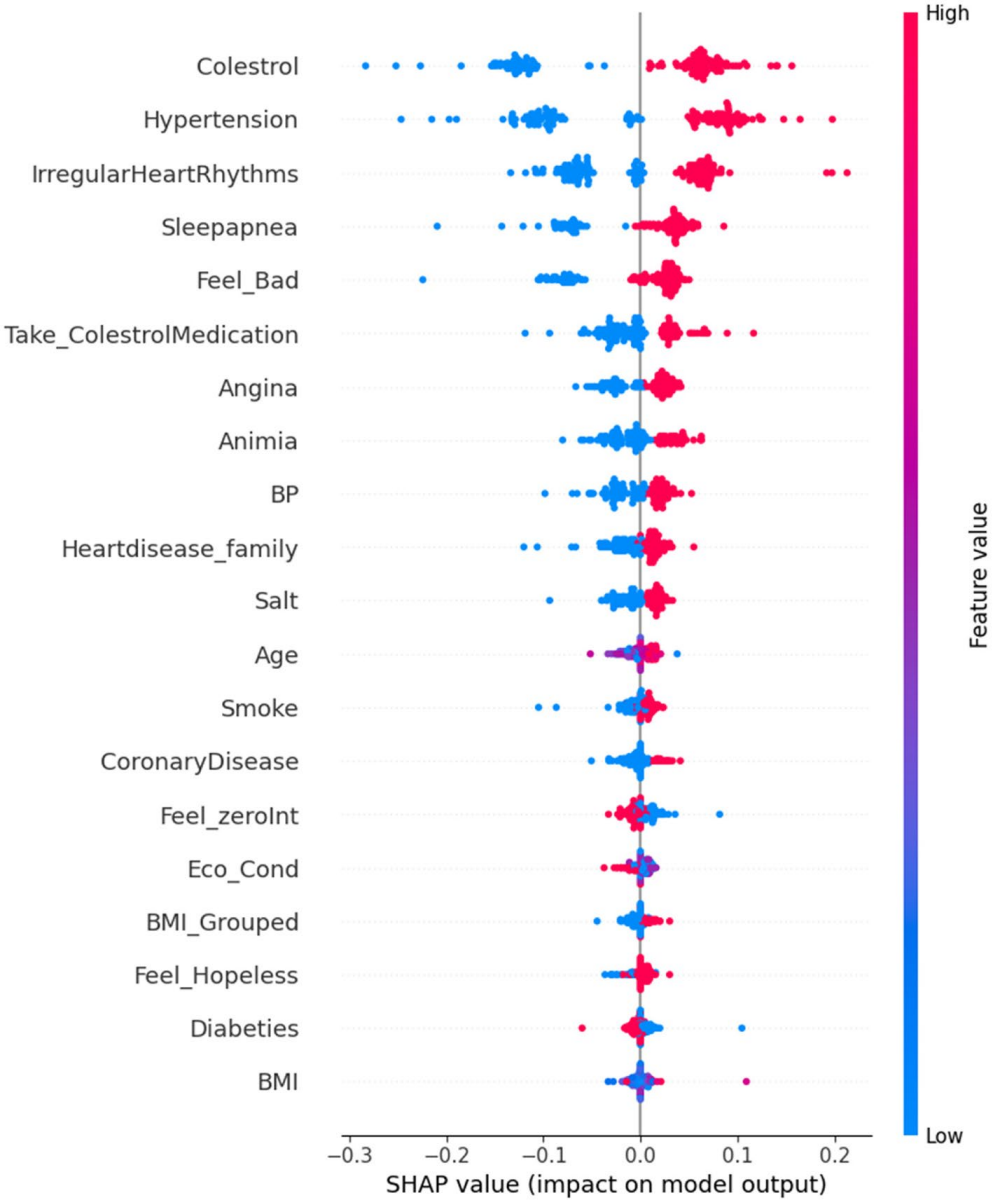
Beeswarm Plot of SHAP values impact on the Random Forest Model

Based on the findings from the aforementioned studies, it is evident that the Random Forest classifier outperforms all other classifiers in terms of predictive accuracy and performance. Therefore, we strongly recommend adopting the Random Forest technique within the system for heart disease prediction. It is important to note that our collected dataset was not previously used for training and testing. Hence, we propose leveraging the best classifier, based on the results presented earlier. It is essential to recognize that the performance of a classifier is not universally superior in all scenarios. It can vary based on factors such as the dataset size and additional attributes. The Random Forest classifier stands out for its robustness in various aspects of model performance and generalization. Unlike decision trees, it is less susceptible to overfitting, making it a reliable choice for modeling complex datasets. Moreover, it can effectively manage noisy or irrelevant features in a dataset without compromising performance. Random Forests demonstrates strong generalization capabilities, allowing it to perform well on unseen data across a wide range of classification tasks. In addition, they can efficiently handle large datasets with high-dimensional feature spaces. This robustness is primarily attributed to their ensemble-based approach, which leverages multiple decision trees to address overfitting and noise and enhance the overall generalization performance. Therefore, while Random Forest demonstrates promise in this context, the choice of the most suitable classifier should always be context-dependent and should be assessed with consideration of the specific data and problem at hand. Hence, we delved into an in-depth analysis of how the features within our dataset influence the outcomes of the Random Forest classifier. To carry out this examination, we harnessed the power of SHapley Additive exPlanations (SHAP). In Fig. 24 and 25, represented as "Bee Swarm Plots" of SHAP values, we gain insight into the effect of every feature on the learned and tested method predictions. Both figures provide a clear visualization of the attributes that significantly influence the output of the model. It is worth noting that among the 28 attributes, only 20 were deemed significant, as shown in the plots. These are the key features that play a pivotal role in shaping the model predictions.

In Fig. 24, the graph displays two axes: the x-axis is designated as "SHAP value (average impact on model output magnitude)," and the y-axis is labeled "Features." The graph clearly illustrates that features with the highest SHAP values encompass cholesterol, hypertension, irregular heart rhythms, and sleep apnea. This implies that these particular attributes are of the utmost importance in influencing the model's predictions. It is worth emphasizing that the SHAP values provided are averages, and the specific impact of a feature on a particular prediction may fluctuate contingent on the values of the other features involved. In Fig. 25, a compelling pattern emerges as we observe the impact of cholesterol values on model predictions. Notably, lower cholesterol values are associated with negative SHAP values, represented by points extending towards the left and becoming increasingly blue. Conversely, higher cholesterol values yielded positive SHAP values, depicted by points extending towards the right and turning increasingly red. The density of these red dots is notably high, indicating that the "Cholesterol" feature exerts a substantial impact on the model's predictions. In essence, the prediction is significantly reliant on the "Cholesterol" feature. Furthermore, it is important to clarify the directional influence of the SHAP values. A positive SHAP score indicates that an attribute elevates the forecast, whereas negative values suggest that a feature has a diminishing effect on the prediction, offering valuable insights into the model's decision-making process. Certain attributes such as "Take_CholesterolMedication," "Angina," and "Anemia" are observed to exert a downward influence on the model's predictions, and this is denoted by the presence of blue dots. Interestingly, " coronary disease appeared to have a comparatively lower impact on the model's predictions. The effects of the other attributes are situated toward the lower portion of the plot, indicating their relatively less significant role in shaping the model's output.

In summary, Figs. 24 and 25 offer a valuable glimpse into the primary determinants that influence the risk of heart disease, as assessed by the Random Forest (RF) model. Nonetheless, it is vital to bear in mind that the SHAP values provided are averages, and that the specific impact of a feature on a particular prediction can vary depending on the values of other associated features. Some supplementary observations were extracted from these figures.

- Individuals with sleep apnea, angina, or a family history of cardiovascular illness have a heightened risk of CVD.
- The adoption of cholesterol medications is correlated with a lower risk of cardiovascular illness.
- Smoking is associated with a higher risk of cardiovascular illness and stroke.
- Experiencing feelings of distress, hopelessness, and lack of interest are also associated with an elevated risk of CVDs.

These data provide a foundation for devising strategies to prevent and mitigate CVD risks. For instance, individuals with elevated cholesterol levels, hypertension, or irregular heart rhythms should collaborate closely with their healthcare providers to manage these conditions effectively. Those with a family history of heart disease or other predisposing risk factors should engage in discussions with their healthcare professionals to explore methods of risk reduction and tailored prevention approaches.

### Efficiency of the RF compared to the other published article

It is observed that in majority cases the efficiency from the RF model is higher as compared to previous similar studies (Table 7).

**Table 7:** Efficiency of the RF compared to the other published article

Paper name	Random forest accuracy
**In our current study**	98.04%
M. I. Hossain et al*.*, “Heart disease prediction using distinct artificial intelligence techniques: performance analysis and comparison,” *Iran J. Comput. Sci.*, 2023, https://doi.org/10.1007/s42044-023-00148-7	97.7%
M. M. Ali, B. K. Paul, K. Ahmed, F. M. Bui, J. M. W. Quinn, and M. A. Moni, “Heart disease prediction using supervised machine learning algorithms: Performance analysis and comparison,” *Comput. Biol. Med.*, vol. 136, no. May, p. 104,672, 2021, https://doi.org/10.1016/j.compbiomed.2021.104672	100%
A. S. S. N. K. Kumar, G. S. Sindhu, D. K. Prashanthi, “‘Analysis and prediction of cardio vascular disease using machine learning classifiers,’ in Proceedings of the 2020 6th International Conference on Advanced Computing and Communication Systems (ICACCS).,” *IEEE*	85.71%
Fahim, K. E., Yassin, H., Amin, M. H., Dewan, P. D., & Islam, A. (2022, September). Detection of Cardiovascular Disease of Patients at an Early Stage Using Machine Learning Algorithms. In *2022 International Conference on Healthcare Engineering (ICHE)* (pp. 1–6). IEEE	73.03%
Hossen, M. A., Tazin, T., Khan, S., Alam, E., Sojib, H. A., Monirujjaman Khan, M., & Alsufyani, A. (2021). Supervised machine learning-based cardiovascular disease analysis and prediction. *Mathematical Problems in Engineering*, *2021*, 1–10	80%
Nashif, S., Raihan, M. R., Islam, M. R., & Imam, M. H. (2018). Heart disease detection by using machine learning algorithms and a real-time cardiovascular health monitoring system. *World Journal of Engineering and Technology*, *6*(4), 854–873	95.76%

## Discussion

Cardiovascular disease (CVD) ranks the highest among all causes of death globally [42]. Late detection of cardiac issues significantly reduces patient prognosis for patients [43]. Machine learning is a vital tool for diagnosing conditions such as heart issues, movement abnormalities, and other disorders. Physicians might gain valuable insights that help them customize each patient's diagnosis and treatment strategy when such information is predicted accurately in advance.

The goal of this project was to predict CVD risk among Bangladeshi people using different machine learning models. In our study, the classifiers Random Forest (96.15%), Decision Tree (96.1%), AdaBoost (94.94%), Naïve Bayes (94.87%), and Bagging Tree (94.87%) have the best precision rates. Among the five techniques studied, random forest was the most accurate. Moreover, the Random Forest classifier maintained the highest balance between correct and incorrect predictions, with an astounding rate of 97.7%. While other models perform admirably, they fall short of random forests. In addition, the Random Forest classifier maintained a strong F1 score, strong recall, and high precision, achieving the highest accuracy of 98.04%. Random Forest produces the best prediction result with 97.7% accuracy, which is similar to previous studies [11]. Similar to our study, a previous study found that the Random Forest (RF) approach achieved almost 100% accuracy, sensitivity, and specificity in identifying features with the highest likelihood of heart disease [42]. Kumar et al. (2020) employed a range of machine learning methods to forecast heart disease [44] and found that, in comparison to alternative classifier methods, the suggested model demonstrated that random forests had the highest accuracy, at 85.71%. Again, in some studies, Naive Bayes achieved the highest accuracy of 84.16% when employing the ten most crucial characteristics [45, 46]. Decision trees have the lowest accuracy rate (77.55%), but when combined with boosting approaches, they outperform with an accuracy of 82.17% [47], according to previous studies. However, with an accuracy of 95.42 %, the Logistic Regression model had the lowest performance. By combining principal component analysis with alternating decision trees, the M.A. Jabbar et al. achieved a 92.2% accuracy using a logistic regression model [48].

The findings of previous studies clearly demonstrate that the Random Forest classifier outperforms all other classifiers in terms of expected accuracy and performance [11]. Incorporating the Random Forest approach into a system for CVD prediction is highly recommended. To validate and forecast cardiovascular illness independently, this study focused on understanding cardiovascular disease and its main contributing factors, in addition to providing a collection of industry-standard benchmark machine learning algorithms. Factors such as salt intake, feelings of inferiority, depression, smoking, blood pressure, family history of heart failure, high cholesterol, anemia, diabetes, hypertension, sleep apnea, and other health issues were also significantly associated with CVD, which is consistent with previous studies [49]. In the current study, the "cholesterol" characteristic played a major role in the forecast. It is also critical to define the direction in which these SHAP values influence each other. A positive SHAP value indicates a feature that increases prediction. Cholesterol is an important risk factor for cardiovascular disorders is cholesterol according to previous studies [50].

This study could impact clinical practice by providing physicians with a new tool to estimate a patient's chance of survival. The results revealed risk factors and subtle trends that may not be readily apparent to medical practitioners. Early identification is critical because quick action can prevent and treat CVD. Machine learning algorithms can be used to calculate a person's lifetime risk of heart disease. These algorithms can enable proactive preventative measures and provide continuous risk assessments by continuously monitoring and analyzing health data.

### Strength

The primary strength of this study lies in its ability to discern the significance and contribution of individual factors to the prediction of cardiovascular disease (CVD) risk, achieved through the utilization of SHAP values. Additionally, this study incorporated both behavioral and clinical factors in the prediction of CVD risk, providing a comprehensive perspective on the influencing variables.

### Limitations

This study has certain limitations. First, it is a cross-sectional study that provides a snapshot of information at a specific point in time. A longitudinal study that tracks patients over an extended period would be beneficial to enhance our understanding and predictive accuracy. Second, the sample size in this study was limited to 651, which may impede the precision of predicting cardiovascular disease (CVD) risk using machine learning models. Future investigations could benefit from larger sample sizes to improve the robustness of our findings.

### Advantages

The findings of this study will be of great assistance to policymakers in making decisions regarding patients with heart failure, especially those who are vulnerable in Bangladesh. Additionally, the proposed best-fitting model, Random Forest, will aid medical professionals and lab technicians in detecting heart failure at an earlier stage of the disease. Furthermore, the government of Bangladesh can utilize this research to gain a better understanding of the current state of heart failure patients and formulate policies in the healthcare sector based on this information. Policymakers can also help with the features that mostly influence the prediction of heart disease.

## Conclusions

This study provides valuable insights into the prediction cardiovascular disease (CVD) in Bangladesh, a country where CVDs are increasingly becoming a leading cause of mortality. Bangladesh has great significance in the study of cardiovascular disease (CVD) because of its effects on the country's socioeconomic development, healthcare infrastructure, and public health. Through the utilization of various machine learning techniques, including Logistic Regression, Naïve Bayes, Decision Tree, AdaBoost, Random Forest, and Bagging Tree classifiers, we aimed to identify the critical factors influencing CVD and develop a robust predictive model. Random Forest was the most successful classifier of the methods examined; it showed the best precision, accuracy, recall, F1 score, and area under the receiver operator characteristic curve (AU-ROC). The Random Forest classifier surpassed other models with a precision rate, providing clinicians with a trustworthy tool for determining patient prognosis and CVD risk. This study highlights the importance of using machine learning techniques in the healthcare industry to improve the early identification and management of CVD, especially in low- and middle-income countries, such as Bangladesh. The way researchers, patients, and healthcare professionals approach the prevention and management of cardiovascular disease is changing significantly owing to the application of machine learning algorithms for CVD prediction. Healthcare practitioners can improve patient care techniques and make better decisions by applying the Random Forest methodology to their clinical practice.

### Supplementary Information

**Supplementary Material 1.**

## Data Availability

Data will be available upon request to corresponding author.

## Abbreviations

(mcL): Platelets per microliter
(mg/dL): Milligram per deciliter
(mmol/L): Millimoles per liter
