# Supplementary material for: Machine learning approach for predicting cardiovascular disease in Bangladesh: evidence from a cross-sectional study in 2023
Source: BMC Cardiovasc Disord. 2024 Apr 18;24:214. doi: 10.1186/s12872-024-03883-2 (PMC11025260; doi:10.1186/s12872-024-03883-2)
Supplement: Supplementary file 1 — Supplementary Material 1. [file 12872_2024_3883_MOESM1_ESM.pdf]

Prediction of survival of patients with heart failure using the best predictive machine learning model

We are conducting a survey. It takes only 3-5 minutes. We can complete the survey If you will Cooperate us by giving information according to the survey.

sorifhossaindhaka@gmail.com Switch account

Not shared

* Indicates required question

Do you have a heart disease according to diagnosis (আপনার কি হৃদরোগ আছে) ? *

- Yes
- No

Age (বয়স) *

Your answer

Gender (লিঙ্গ) *

- Male
- Female

Height (উচ্চতা) *

Your answer

Weight (ওজন) *

Your answer

Education (শিক্ষা) *

- Primary
- Secondary
- Higher secondary
- No education

Division (বিভাগ) *

- Dhaka
- Chattogram
- Khulna
- Rajshahi
- Barishal
- Sylhet
- Mymensingh
- Rangpur

Socioeconomic status (আর্থ - সামাজিক অবস্থা) . (Income/tk) *

- <20 thousand
- 21-40 thousand
- > 40 thousand

Residence (বাসস্থান) *

- Urban
- Rural

Do you take physical exercise regularly (আপনি কি নিয়মিত শারীরিক ব্যায়াম করেন) ? *

- Yes
- No
- Sometimes

Do you have a sound sleep at night (আপনার কি রাতে ভালো ঘুম হয়) ? *

- Yes
- No
- Sometimes

Do you consume two or more servings of fruits or vegetables per day (আপনি কি প্রতিদিন দুই বা তার বেশি ফল বা শাকসবজি খান) ? *

- Yes
- No
- Sometimes

Do you eat junk food regularly (আপনি কি নিয়মিত জাঙ্ক ফুড খান) ? *

- yes
- No
- Sometimes

Do you keep too much salt in your diet (আপনি কি আপনার খাদ্যতালিকায় অতিরিক্ত লবণ রাখেন) ? *

- Yes
- No
- Sometimes

Do you feel bad about yourself (তোমার কি নিজের খারাপ লাগছে) ? *

- Yes
- No
- Sometimes

Do you feel no interest or pleasure in doing any things (আপনি কি কোন কাজ করতে কোন আগ্রহ বা আনন্দ অনুভব করেন না) ? *

- Yes
- No
- Sometimes

Do you feel hopeless (আপনি কি আশাহীন বোধ করেন) ? *

- Yes
- No
- Sometimes

Do you have smoking habit (আপনার কি ধূমপানের অভ্যাস আছে) ? *

- Yes
- No
- Sometimes

Do you have the habit of drinking alcohol (আপনার কি মদ পান করার অভ্যাস আছে) ? *

- Yes
- No
- Sometimes

Do you have the presence of high cholesterol level according to diagnosis (আপনার কি উচ্চ কোলেস্টেরলের উপস্থিতি আছে) ? *

- Yes
- No

Do you have blood pressure according to diagnosis (আপনার কি রক্তচাপ আছে) ? *

- Yes
- No

Do you have any family history of heart failure disease (আপনার কি হৃদরোগের কোনো পারিবারিক ইতিহাস আছে) ? *

- Yes
- No

Do you have the presence of anemia according to diagnosis (আপনার কি রক্তশূন্যতার উপস্থিতি আছে) ? *

- Yes
- No

Do you have any type of diabetes according to diagnosis (আপনার কি কোন ধরনের ডায়াবেটিস আছে) ? *

- Yes
- No

Do you have the presence of hypertension according to diagnosis (আপনার কি উচ্চ রক্তচাপের উপস্থিতি আছে) ? *

- Yes
- No

Do you have sleep apnea problem (আপনার কি নিদ্রাহীনতার সমস্যা আছে) ? *

- Yes
- No

Do you have irregular heart rhythms according to diagnosis (আপনার কি হৃদপিন্ডের অনিয়মিত ছন্দ আছে) ? *

- Yes
- No

Do you have coronary artery disease according to diagnosis (আপনার কি হৃদপিণ্ডে ধমনীর অসুখ আছে) ? *

- Yes
- No

Do you have angina symptoms according to diagnosis (আপনার কি অ্যানজিনা বা বুকে ব্যথার উপসর্গ আছে) ? *

- Yes
- No

Do you have kidney, lungs or others major disease according to diagnosis (আপনার কি কিডনি, ফুসফুস বা অন্য কোন বড় রোগ আছে) ? *

- Yes
- No

Do you take Statin to decrease Cholesterol level.(আপনি কি কোলেস্টেরলের মাত্রা কমাতে স্ট্যাটিন খান) ? *

- Yes
- No
- Sometimes

Ejection fraction/ Percentage of blood leaving the heart at each contraction (2-7)min

Your answer

Platelets in the blood in kilo platelets\ ml (150000-400000)cumm

Your answer

Level of creatinine in blood in mg\dl (0.40-1.40)

Your answer

Level of sodium in blood in mmol\L (135-148)

Your answer

Submit

Clear form

Never submit passwords through Google Forms.

This content is neither created nor endorsed by Google. Report Abuse - Terms of Service - Privacy Policy

Google Forms
